# Reliable measures of rest-activity rhythm fragmentation: how many days are needed?

**DOI:** 10.1186/s11556-024-00364-5

**Published:** 2024-10-19

**Authors:** Ian Meneghel Danilevicz, Sam Vidil, Benjamin Landré, Aline Dugravot, Vincent Theodor van Hees, Séverine Sabia

**Affiliations:** 1 Epidemiology of Ageing and Neurodegenerative Diseases, Université Paris Cité, INSERM, U1153, CRESS, 10 Avenue de Verdun, Paris, 75010 France; 2 Accelting, Almere, The Netherlands; 3 UCL Brain Sciences, Division of Psychiatry, University College London, London, UK

**Keywords:** Non-wear, Imputation, Transition probability, Simulation, Intradaily variability, Inter-daily stability

## Abstract

**Background:**

A more fragmented, less stable rest-activity rhythm (RAR) is emerging as a risk factor for health. Accelerometer devices are increasingly used to measure RAR fragmentation using metrics such as inter-daily stability (IS), intradaily variability (IV), transition probabilities (TP), self-similarity parameter (α), and activity balance index (ABI). These metrics were proposed in the context of long period of wear but, in real life, non-wear might introduce measurement bias. This study aims to determine the minimum number of valid days to obtain reliable fragmentation metrics.

**Methods:**

Wrist-worn accelerometer data were drawn from the Whitehall accelerometer sub-study (age: 60 to 83 years) to simulate different non-wear patterns. Pseudo-simulated data with different numbers of valid days (one to seven), defined as < 1/3 of non-wear during both day and night periods, and with omission or imputation of non-wear periods were compared against complete data using intraclass correlation coefficient (ICC) and mean absolute percent error (MAPE).

**Results:**

Five days with valid data (97.8% of participants) and omission of non-wear periods allowed an ICC ≥ 0.75 and MAPE ≤ 15%, acceptable cut points for reliability, for IS and ABI; this number was lower for TPs (two-three days), α and IV (four days). Overall, imputation of data did not provide better estimates. Findings were consistent across age and sex groups.

**Conclusions:**

The number of days of wrist accelerometer data with at least 2/3 of wear time for both day and night periods varies from two (TPs) to five (IS, ABI) days for reliable RAR measures among older adults.

**Supplementary Information:**

The online version contains supplementary material available at 10.1186/s11556-024-00364-5.

## Background

Accelerometer devices provide objective and continuous measurements of human movement behavior [1] and are increasingly used to derive metrics of fragmentation of rest-activity rhythm (RAR), such as inter-daily stability (IS) [2–4], intradaily variability (IV) [2–4], 3) transition probability (TP) between rest and activity during the day and between sleep and wake during the night [4–6], and detrended fluctuation analysis (DFA)-derived self-similarity index (named alpha) [7–9] and activity balance index (ABI) [4]. There is emerging evidence of a more fragmented and less stable RAR to be associated with higher risk of adverse health outcomes [10], specially in older adults [11, 12], including motor [13], vascular [14, 15] and neurodegenerative [12, 16–18] health outcomes, independently of total duration of physical activity [13, 18] or sleep [19].

In this context, it is important to ensure the measure of RAR fragmentation metrics, originally developed in the context of long period of continuous wear without removal of the accelerometer [20], to be reliable in population studies. Indeed, non-wear periods exist in real life data and there is a need to evaluate the extent to which partial data (that is including non-wear periods) can be used to reduce selection bias in studies examining the association between these metrics and health. A recent review of the literature on practices in the accelerometer field reported that four days is commonly used as the minimum number of days of wear (75% of papers using waist device adopted this criterion and 59% of the ones using wrist device) and 10 h of daily wear time as the minimum to consider a day to be valid (88% for waist and 42% for wrist devices) [21]. This practice is supported by studies investigating the reliability of variables such as total time of physical activity and sedentary behavior using intraclass correlation coefficient (ICC), a metric to evaluate the reliability of measurements. Overall, in these studies, the minimum number of valid days ranges between three [22–24] and six days [25].

For RAR fragmentation metrics, there is little research on the impact of the number of valid days and the definition of a valid day (based on the proportion of non-wear) on their reliability. One study evaluated the impact of the number of days of wear on IS and IV, and found IV estimations to be more robust to non-wear periods than IS and imputation of non-wear periods equal to or longer than one day unsatisfactory [26]. Outside the realm of accelerometer data, few studies investigated the robustness of the DFA to missing data [27, 28] and showed alpha to be robust to missing data even in small time series whether or not missing data were imputed. To the best of our knowledge, no previous study investigated the properties of TP in regards to non-wear period. Furthermore, the impact of the definition of a valid day being based on the proportion of non-wear separately over day and night periods or over an entire day has not been investigated. Finally, two main strategies to deal with non-wear periods are to omit or impute data during these periods. Several imputation techniques have been proposed [26–28], but there is no clear evidence of simple or more sophisticated methods to perform better, the critical point being proceeding with an imputation at the epoch level [23, 29, 30].

In order to overcome current gaps in the literature on the impact of non-wear on accelerometer measures of RAR fragmentation, this study aims to determine the minimum number of valid days, using three different definitions of a valid day, for reliable measures of these metrics. A secondary objective was to assess whether there is any advantage to impute rather than omit non-wear periods.

## Methods

### Study population

The Whitehall II study is an ongoing prospective cohort study established in 1985–1988 among 10,308 British civil servants with clinical examinations every four-five years since inception. Written informed consent for participation was obtained at each contact. Research ethics approval was obtained from the University College London ethics committee (latest reference number 85/0938). The Whitehall II accelerometry substudy was conducted during the 2012–2013 wave of data collection (age range: 60–83 years) for participants seen at the London clinic and those living in the south-eastern regions of England who underwent clinical examination at home. It was among the first large studies in adults to use wrist-worn devices recording raw accelerometry over three axes, along with the UK Biobank (2013–2016) [23], the NHANES (2011–2014) [31], and the Women’s Health Accelerometry collaboration (2011–2014) [32], with the Whitehall II accelerometer substudy being specific to older adults.

### Accelerometer measurement

Participants were requested to wear a tri-axial accelerometer (GENEActiv Original; Activinsights Ltd, Kimbolton, United Kingdom) on their non-dominant wrist for nine consecutive 24-hour days. Accelerometer data, sampled at 85.7 Hz and expressed relative to gravity (1*g* = 9.81 m/s^2^), were processed using **GGIR** R package (2.9-0) [33]. The Euclidean norm minus one of raw acceleration was calculated and corrected for calibration error. Sleep episodes were identified using a validated algorithm guided by a sleep log (daily report of sleep onset and waking time) [34]. Data from waking onset on day 2 to same time on day 8 were retained, resulting in 7 days of data. The day period was defined as the period from wake up to start the day to sleep onset at night and night period as the one from sleep onset at night to the following wake up to start the day. An entire day was defined from waking onset to the next waking onset. Non-wear time was detected using a previously described algorithm [35]. Average acceleration over 60s-epoch lower than 40 milligravity (m*g*) was classified as *rest* corresponding to activities not classified as light or moderate-to-vigorous activities [36, 37].

### Rest-activity rhythm (RAR) fragmentation metrics

The following RAR fragmentation metrics were considered: IS, a measure of the consistency of the rhythm across days [2–4]; IV, an index of hourly fragmentation of the rhythm [2–4]; TPs, probabilities of state change from wake to sleep (TP_ws, n_) and sleep to wake (TP_sw, n_) during the night, and from rest to activity (TP_ra, d_) and activity to rest (TP_ar, d_) during the day [4–6]; alpha, a measure of the fractal nature and stationarity of a time series [9]; and ABI, a measure of the fractal nature of a time series [4]. For more details on the calculation of these metrics, see supplementary Methods.

### Simulation methodology

After exploring non-wear time patterns among participants with non-wear episodes during the observation period, we conducted a pseudo-simulation study to examine how many valid days are needed to have reliable estimates of RAR fragmentation metrics. Using data from individuals with complete data over 7 days, we artificially created 20 simulated batches corresponding to three scenarios for the definition of a valid day, with for each scenario, the number of valid days varying from one to seven, and with omission or imputation of non-wear periods. The scenarios are:

- **Scenario 1: A valid day is defined as the accelerometer worn at least 2/3 of the entire day**. To achieve this, a block of non-wear time is included in the day artificially, and its length is drawn following a Uniform distribution between one epoch and 1/3 of the wear time of that day. Then, the time location of the block in the day is drawn following a Uniform distribution between the first epoch of the day and the last minus the length of the block. Consequently, the block can be allocated in any period of the day (morning, afternoon or night).
- **Scenario 2**: **A valid day is defined as the accelerometer worn at least 2/3 of both the day and night periods**. To achieve this, two blocks of non-wear time are included in the day artificially, and their lengths are drawn following two independent Uniform distributions between one epoch and 1/3 of the wear time of that awake period and sleep period, respectively. Then, the time location of each block in the day is drawn following a Uniform distribution between the first epoch of the day/night period and the last minus the length of the block. Consequently, the blocks are allocated in separate periods of the day (usually, the first block is in the morning or afternoon, and the second at night).
- **Scenario 3: A valid day is defined as the accelerometer worn full time during the entire day**.

**Fig. 1:**
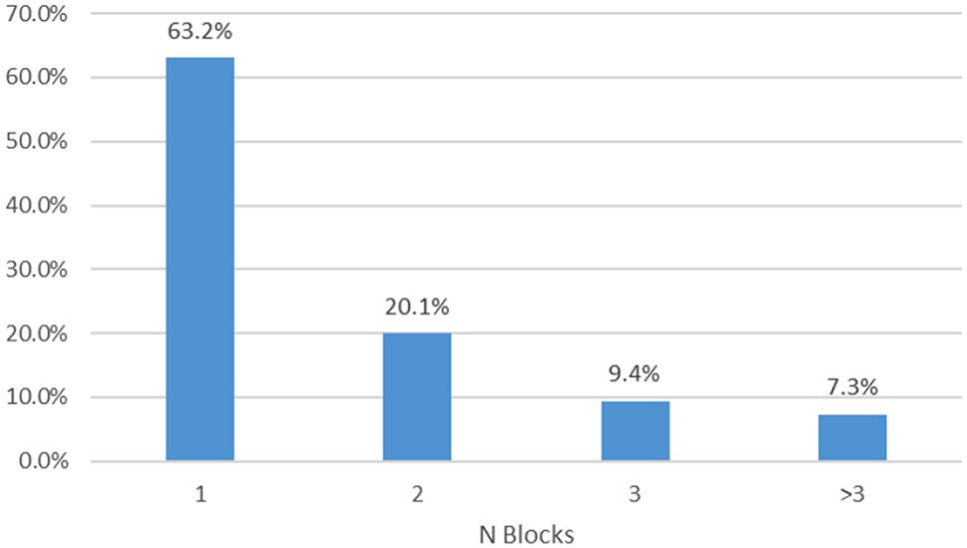
Distribution of N blocks of non-wear over the full observation period among the 1241 participants with at least one non-wear event. *Note* % of non-wear per person-day **(a)** was calculated among the 219 person-days with at least one non-wear episode and less than 1/3 of non-wear during the day period and % of non-wear per person-night **(b)** was calculated among the 1490 person-nights with at least one non-wear episode and less than 1/3 of non-wear during the night period

In Scenario 1 and 2, we chose to define a valid day based on at least 2/3 of wear time, either during the entire day or separately during the day and night periods, as it is similar to what is commonly done in the literature (10 h of wear during waking hours [21] or 2/3 of 24 h [38]), particularly in physical activity research, where most of the studies using accelerometer data stands. These scenarios with blocks of non-wear time rather than random epochs of non-wear were chosen to mimic what was observed in the population study. In total, among the 1241 participants of the Whitehall accelerometer substudy with at least one episode of non-wear, 63.2%, 20.1%, and 9.4% had one, two, and three non-wear periods, respectively, over the overall observation period (Fig. 1). In the simulation, Scenario 1 allows one bout of non-wear per day and Scenario 2 two bouts per day to account for non-wear during the day and the night. Further, both scenarios have the same expected average of non-wear time (16.4%) within a valid day, because each block’s length follows a Uniform distribution between one epoch and 1/3 of the period.

The cross of three scenarios and seven numbers of days retained (1, 2, 3, …, 7) resulted in 20 batches of simulation, Scenario 3 with seven days being the reference. Each batch included 2859 participants for which the eight metrics (IS, IV, four TPs, alpha and ABI) were estimated twice, omitting or imputing non-wear periods. Omitting means that non-wear periods were excluded from the signal to calculate the metrics. The imputation method consisted of taking the average at the epoch level of the equivalent moment on the other days when the device was worn [23, 35]. For IS, the minimum number of days that could be tested was three as in order to be calculated this estimate needs that each hour of the day is observed at least twice during the observation period.

The reliability of the eight metrics was estimated using the ICC, a measure of correlation between the simulated batches and the reference batch (seven days of complete data), and mean absolute percent error (MAPE), a robust metric that summarizes the differences between the simulated batches and the reference [39]; see supplementary methods for details on the calculation. An ICC ≥ 0.75 and a MAPE ≤ 15% were considered acceptable [40, 41]. Given that the TP metrics are day and night dependent, we chose to present findings for Scenario 2 in the manuscript and those for Scenario 1 and 3 in the supplementary material.

Sensitivity analyses were conducted to examine whether findings for Scenario 2 were similar as a function of sex or age (< 70 years, ≥ 70 years). In post-hoc analyses, we also illustrated the impact of imputation on the signal of a selected participant.

All fragmentation metrics presented here were calculated using R software and the codes are available on GitHub (https://github.com/iandanilevicz/frag_metrics). They will be included in a future release of **GGIR** R package (> 3.1–4) [42], and alpha and ABI can be calculated using **DFA** R package (1.0–0) [43].

## Results

Among the 4880 participants invited to the accelerometer sub-study at the 2012–2013 wave of data collection, 4267 returned the device among whom 4106 had data that could be analysed (flowchart in Supplementary Fig. 1). The number of participants with a minimum number of valid days varied according to the definition of a valid day. Table 1 shows that 3867 individuals wore the accelerometer for more than 2/3 of the entire day (wake up to next wake up) over seven days, 3832 for both 2/3 of the day (wake up to sleep onset) and 2/3 of the night (sleep onset to next wake up) periods, and 2859 wore it full time over seven days. These numbers increase as the number of valid days decreases. Even though 2859 individuals represent a large part of the population (70%), the demographic characteristics, such as the average age, mean body mass index, sex proportion, and married/cohabitation percentage, are significantly different from those who worn the devices for a shorter period (supplementary Table 1).

**Table 1:** Number of participants meeting valid day requirements according to valid day definitions

*N* valid days	Scenario 1: Accelerometer wear time ≥ 2/3 of the entire day*	%	Scenario 2: Accelerometer wear time ≥ 2/3 of both day and night periods	%	Scenario 3: Accelerometer worn during the entire day	%
1	4100	99.9	4097	99.8	4093	99.7
2	4093	99.7	4084	99.5	4064	99.0
3	4074	99.3	4067	99.1	4034	98.3
4	4054	98.8	4046	98.6	3978	96.9
5	4020	98.0	4012	97.8	3854	93.9
6	3985	97.1	3974	96.8	3571	87.0
7	3867	94.2	3832	93.4	2859	69.7

* An entire day is defined as the period between wake up to next wake up (it combines the day (wake up to sleep onset) and night (sleep onset to wake up) periods)

**Fig. 2:**
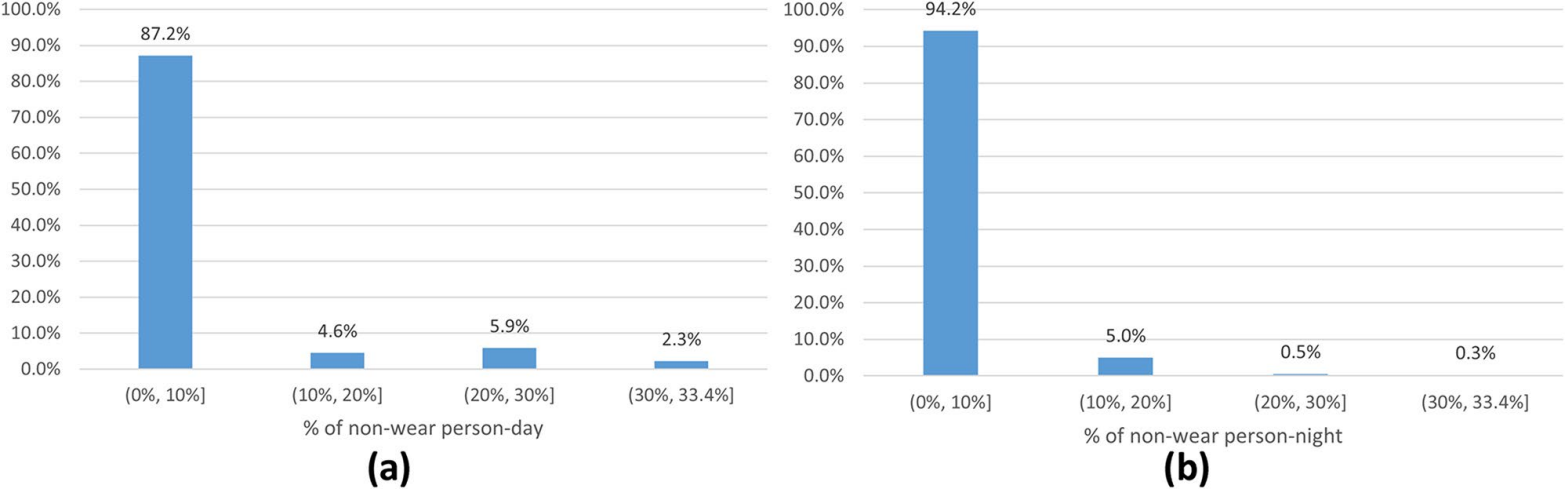
Distribution of % of non-wear among valid day and night periods. *Note* % of non-wear per person-day **(a)** was calculated among the 219 person-days with at least one non-wear episode and less than 1/3 of non-wear during the day period and % of non-wear per person-night **(b)** was calculated among the 1490 person-nights with at least one non-wear episode and less than 1/3 of non-wear during the night period

The non-wear episodes are not frequent: in the 1241 individuals with at least one event of non-wear, these episodes occur mainly once (63.2%) or twice (20.1%) over the full observation period (Fig. 1). Furthermore, the non-wear episodes are brief in the majority of cases, they are shorter than 10% of the day or night period duration (87.2% and 94.2%, respectively) for which participants removed the accelerometer for less than 1/3 of the respective periods (Fig. 2).

**Fig. 3:**
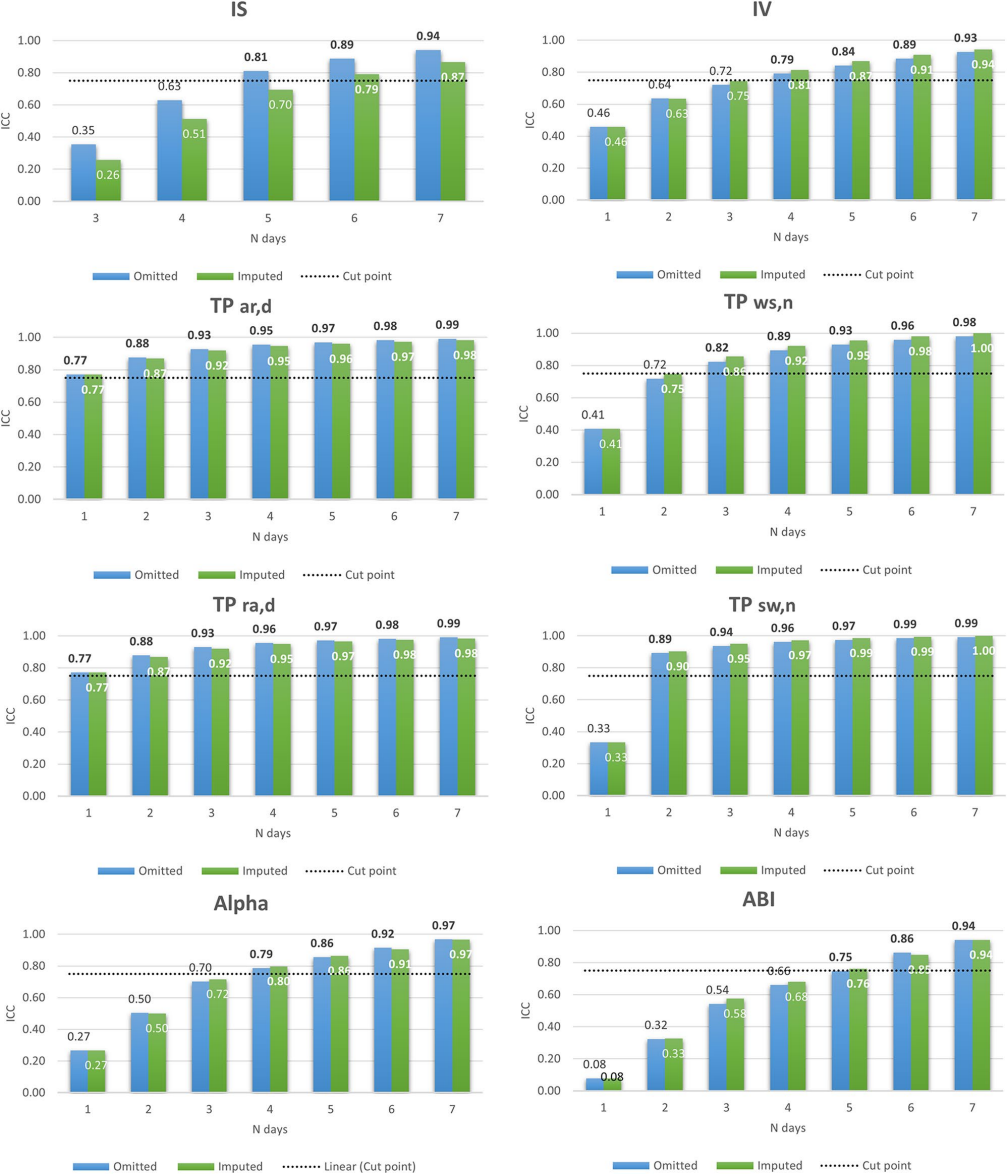
ICC according to the number of valid days defined as accelerometer wear time ≥ 2/3 of both day and night periods (Scenario 2)*. Abbreviations: intraclass correlation coefficient (ICC), inter-daily stability (IS), intradaily variability (IV), transition probability (TP), TP from activity to rest during the day (TP_ar, d_), TP from wake to sleep during the night (TP_ws, n_), TP from rest to activity during the day (TP_ra, d_), TP from sleep to wake during the night (TP_sw, n_), and activity balance index (ABI). Bold values correspond to ICC ≥ 0.75

In the simulation study, the estimation of the fragmentation metrics (IS, IV, TP, alpha and ABI) of the reference population (2859 who worn the accelerometer full time during 7 days) was compared with the one of the other 20 batches. Figure 3 displays the ICC for the metrics in case of Scenario 2, where a valid day is defined as accelerometer wear time ≥ 2/3 of both day and night periods, omitting (blue) or imputing (green) non-wear periods. When non-wear periods were omitted, the ICC was slightly better for IS, TP_ar, d_ and TP_ra, d_, while imputation of non-wear periods tended to result in better ICC for IV, TP_ws, n_ and TP_sw, n_. Using the criterion of ICC ≥ 0.75, a reliability estimation was achieved with at least five days for ABI and IS, four days for IV and alpha, one day for TPs related to the day period, two to three nights for TPs related to the night period when non-wear period were omitted. Similar findings were observed when non-wear periods were imputed, except for IS (which required an extra day). Results for Scenario 1 (valid day defined as device worn at least 2/3 of the entire day) and 3 (device not removed at all during the entire day) are available in the Supplementary Figs. 2 and 3. The ICC for Scenario 1 was slightly worse than for Scenario 2 (in 28 out 40 cases when non-wear periods were omitted, and in 33/40 cases when imputing non-wear periods. Still, there was little impact on the number of valid days needed using the criterion of ICC ≥ 0.75. The ICC for Scenario 3 was slightly better than for Scenario 2. There was a moderate impact on the number of valid days needed (one day less for IS, IV, and ABI when non-wear periods were omitted, and similarly for IS and IV when non-wear periods were imputed).

**Fig. 4:**
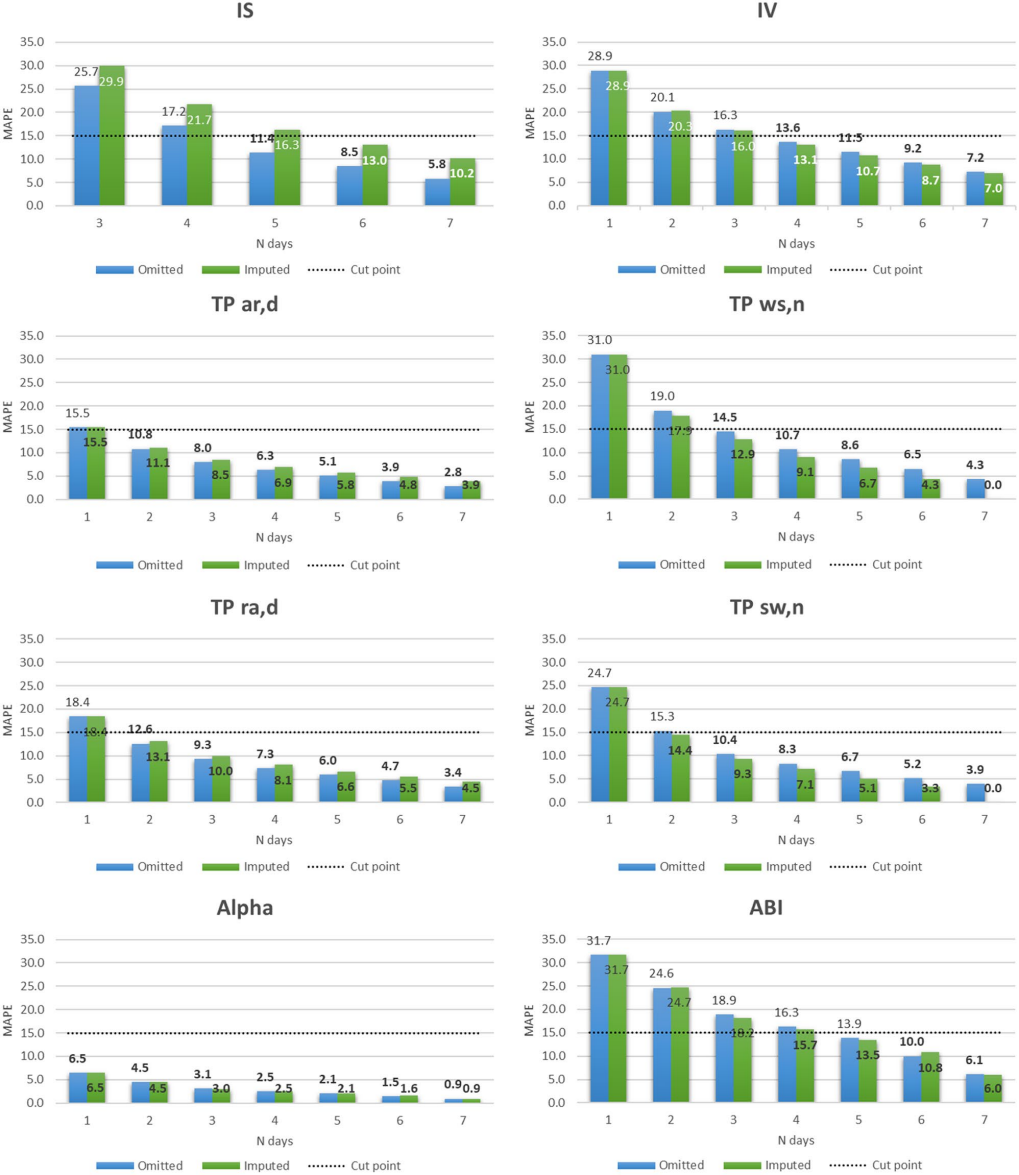
MAPE according to the number of valid days defined as accelerometer wear time ≥ 2/3 of both day and night periods (Scenario 2)*. Abbreviations: mean absolute percent error (MAPE), inter-daily stability (IS), intradaily variability (IV), transition probability (TP), TP from activity to rest during the day (TP_ar, d_), TP from wake to sleep during the night (TP_ws, n_), TP from rest to activity during the day (TP_ra, d_), TP from sleep to wake during the night (TP_sw, n_), and activity balance index (ABI). Bold values correspond to MAPE ≤ 15%

When using the MAPE as the performance measure, in Scenario 2 for valid day definition, we found that at least five days were needed to achieve a MAPE ≤ 15% for IS and ABI, four days for IV, two days for TPs related to the day period, two-to-three nights for TPs related to the night period and one day for alpha when non-wear periods were omitted (Fig. 4). MAPE values tended to be slightly better when imputing the non-wear periods for IS, TP_ar, d_ and TP_ra, d_, but the reverse was true for IV, TP_ws, n_, TP_sw, n_, alpha and ABI. Results for Scenarios 1 and 3 are available in the Supplementary Figs. 4 and 5 and show similar results to ICC. MAPE values for Scenario 1 were slightly worse than Scenario 2, but without changing decisions on the number of days needed, while for Scenario 3 they were slightly better than for Scenario 2 with one day less found to be needed for IS, IV, and ABI whether non-wear periods were omitted or imputed. Sensitivity analyses show findings to be substantially the same for men and women (Supplementary Figs. 6 and 7 for ICC and 8 and 9 for MAPE), and younger and older age groups (Supplementary Fig. 10 for ICC and 11 for MAPE in those aged < 70 years, Supplementary Fig. 12 for ICC and 13 for MAPE in those aged ≥ 70 years). The only exception is for ABI, which requires six days for older age group and women when considering ICC.

In post-hoc analyses, in order to understand the impact of imputation on the fragmentation metrics, we plotted the observed accelerometry signal as a function of time of the day for six days for one individual and its average, we also displayed one specific day, removed four hours (simulated non-wear period), and imputed these hours based on the mean of the other days (Supplementary Fig. 14). The imputed hours are smoother (less variability and more similarity) than the original signal over these hours. Some RAR fragmentation metrics might be affected by this imputation procedure explaining why for example the reliability of IS (a measure of similarity) is lower when non-wear periods are imputed.

## Discussion

This paper aimed to determine the minimum number of valid days of accelerometer data for reliable metrics of RAR fragmentation. Simulated scenarios were proposed to mimic natural human behavior of non-wear time using real accelerometer data among more than 2800 older adults. This study presents two key findings. One, we found that five days of accelerometer wear with no more than 1/3 of non-wear during both day and night periods provided reliable estimates of all fragmentation metrics; this number of required days varies as a function of the metric considered, between two-to-three days for TPs, four for IV and alpha, and five for IS and ABI. Two, whether non-wear time was imputed or omitted had little impact on the findings.

In this paper, we examined the impact of the number of valid days of accelerometer wear on RAR fragmentation metrics using three different definitions of a valid day. These definitions were chosen based on the literature and practical aspects. Most research on the impact of non-wear time in accelerometry field has focussed on physical activity during day time and a common approach is to define a day as valid if the device is worn for at least 10 h during the waking time, corresponding to approximatively 2/3 of the waking periods [21]. Given that the fragmentation metrics cover either the entire day (such as IS or IV) or are specific to day or night periods (such as the TP), we chose two scenarios (1 and 2) that were specific either to the entire day or the day/night periods while the third scenario was the most stringent requiring the device to be worn over the entire day.

Overall, we found that five days are enough to reliably estimate all fragmentation metrics under the scenario where a valid day is defined as at least 2/3 of wear during both the day and night periods and non-wear periods are omitted, this was the case both in men and women and in the younger (60–70 years) and older (70–83 years) age groups, except for ABI which requires six days for women and the oldest age group. This is in line with the recommended minimum of five days of observations in the nparACT software package to calculate IS and IV, although no recommendation was made regarding the definition of a valid day [44]. This requires one day more than the four days of wear commonly used in studies on accelerometer-assessed physical activity [21] although findings on the recommended number of valid days varies between studies with for example studies reporting the minimum number of days of wear should be three [22–24], four [45], and six [25] days. The majority of the cited studies used seven days as the total number of days for reference [22–24, 45], except the last one, which uses fourteen days [25]. Given the lack of guidelines on the number of days required for reliable measures of RAR metrics, there is heterogeneity in the previous studies using these metrics, studies requiring between four to seven days for IS and IV [46], and between three to seven days for fragmentation index [47].

Our findings differ according to the fragmentation metrics used, with the number of required days varying between two-three for TPs, four for alpha and IV, and five for IS and ABI. IS and ABI were more impacted by non-wear periods than IV, the self-similarity parameter alpha and TPs. A previous study also reported the impact of non-wear to be higher for IS than IV [26]. In their original study on IS and IV, van Somoren and colleagues recommended whenever possible a full week of accelerometry data [20]. This was confirmed in a further study where they recommended one week or more for IS and between three and six days for IV using ICC ≥ 0.7 among patients with insomnia or dementia [48]. Considering the DFA analysis, the MAPE for self-similarity parameter alpha suggests only a 6.5% difference between one and seven days of observations. This is in line with other scientific domains, such as genetics [49], respiratory function [50] and geology [28, 51], where a vector of length of one thousand has been reported to be sufficient to provide robust estimates. To the best of our knowledge, no previous study has investigated the impact of non-wear for TPs, the closest study was on a sleep fragmentation index that suggested three days were required for an ICC of 0.7 [25], which is in line with our findings.

There was no large difference in the ICC and MAPE whether the non-wear periods were omitted or imputed, apart for the self-similarity parameter alpha and IS. For alpha, the ICC tend to suggest that four days of data are required for reliable measures while the MAPE suggests one day to be enough, irrespective of omission or imputation of non-wear period. The main difference relative to IS relates to the differences in the treatment of non-wear periods so that imputation of non-wear periods tends to worsen both ICC and MAPE. As illustrated in our example, imputation tends to smooth the signal and reduce differences between days leading to higher IS as compared to the reference signal. This is in line with van Somoren and colleagues’ recommendations to omit non-wear periods when calculating IS and IV [20]. Some researchers might prefer to impute or not non-wear periods in their sample depending on the considered metrics. In this study, we imputed non-wear periods at the epoch level using the mean from other available days at the same time of the day. This approach is commonly used to analyse accelerometer data [23, 35], particularly in the context of physical activity. Other methods have been used in the accelerometry field [26] but there was no evidence of these methods to perform better [26–28]. Future studies may evaluate whether other imputation methods for non-wear periods might reduce the impact of non-wear on fragmentation metrics.

We found only a little impact of the definition of a valid day on the minimum number of days required to have reliable fragmentation metrics. Keeping a balance between wear during the day and night periods has only a small added value to the performance indices. Overall, the minimum number of days required to reach an ICC ≥ 0.75 and a MAPE ≤ 15% was similar in scenarios splitting (Scenario 2) or not (Scenario 1) the non-wear time between day and night periods. As expected, performance indices were better in Scenario 3 defining a valid day as a day with no non-wear time although the minimum number of days to have all fragmentation metrics with an ICC ≥ 0.75 and a MAPE ≤ 15% remained five. In the Whitehall II accelerometer study, using five days as the minimum number of valid days resulted in retaining 98.0%, 97.8% and 93.9% of the sample for Scenario 1, 2, and 3 respectively compared to 69.7% of the sample who worn the device for the entire seven-day period. In a more general context of physical activity and sleep research, retaining five days with at least 2/3 of wear time during both day and night periods appears to be a reasonable compromise between reliability of the measures and reduced selection bias [29].

This study has several strengths that include (1) its large sample size, (2) the use of MAPE, a reliability performance index robust to outliers, in addition to the more commonly used ICC, (3) the simulation plan that emulates natural patterns of non-wear blocks, (4) the use of tri-axial raw accelerometry devices as commonly used nowadays and over the last decade since their inception, and (5) to our knowledge, the first evaluation of the impact of non-wear for TPs and self-similarity alpha metrics using accelerometer data. Findings need to be considered in light of some limitations: (1) only one imputation method was applied, and future studies should investigate whether other methods provide better performance, (2) we chose to simulate one block on non-wear per day or night periods based on observation of non-wear patterns in our study population, but scenario using more blocks of non-wear might be relevant to be studied for example for non-waterproof devices as they are more likely to be removed several times per day unlike the device used in the present study, (3) although simulations were based on real life data, findings might be specific to the studied population (older adults) and accelerometer wear protocol; further studies should replicate this work in different population settings (younger adults, clinical population, and other ethnic groups) and for several accelerometer placements and brands.

## Conclusions

This study shows that the number of valid days needed for reliable RAR measures varies as a function of the metrics considered. Overall, five days of wear of wrist accelerometer, allowing no more than 1/3 of non-wear for each day and night periods, allow for reliable measures of fragmentation metrics (IS, IV, four TPs, alpha and ABI) while retaining a large proportion of the sample in the analysis. The treatment of non-wear periods (omitted or imputed) had little impact on the findings. These findings applied to both men and women, and to the youngest (60–70 years) and oldest (70–83 years) age groups. If researchers have less than five days of observations, then the analysis of RAR fragmentation metrics could be restricted to TPs, IV and the self-similarity parameter alpha, as these metrics require between two and four days of data to provide reliable measures.

## Electronic supplementary material

Below is the link to the electronic supplementary material.

Supplementary Material 1

## Data Availability

Data, protocols, and other metadata of the Whitehall II study are available to the scientific community via the Whitehall II study data sharing portal (https://www.ucl.ac.uk/epidemiology-health-care/research/epidemiology-and-public-health/research/whitehall-ii/data-sharing). All metrics discussed here were calculated using R software and the codes are available on GitHub (https://github.com/iandanilevicz/frag_metrics) and will be included in the next version of GGIR R package (https://cran.r-project.org/web/packages/GGIR/index.html).

## Abbreviations

ABI: Activity balance index
DFA: Detrend fluctuation analysis
ICC: Intraclass correlation coefficient
IS: Inter-daily stability
IV: Intradaily variability
MAPE: Mean absolute percent error
RAR: Rest-activity rhythm
TP: Transition probability
