# Supplementary material for: Reliable measures of rest-activity rhythm fragmentation: how many days are needed?
Source: Eur Rev Aging Phys Act. 2024 Oct 19;21:29. doi: 10.1186/s11556-024-00364-5 (PMC11490056; doi:10.1186/s11556-024-00364-5)
Supplement: Supplementary file 1 — Supplementary Material 1 [file 11556_2024_364_MOESM1_ESM.docx]

Supplementary material of “Reliable measures of rest-activity rhythm fragmentation: how many days are needed?”

Table of contents

[Methods Rest-activity rhythm (RAR) fragmentation metrics 3](#_Toc171350833)

[Methods to compare results from pseudo-simulation 4](#_Toc171350834)

[Table 1 Sample characteristics by selection criteria in the Whitehall II accelerometer substudy 5](#_Toc171350835)

[Figure 1 Study flowchart 6](#_Toc171350836)

[Figure 2 ICC according to the number of valid days defined as accelerometer wear time ≥ 2/3 of the entire day* (Scenario 1) 7](#_Toc171350838)

[Figure 3 ICC according to the number of valid days defined as accelerometer worn during the entire day* (Scenario 3) 8](#_Toc171350839)

[Figure 4 MAPE according to the number of valid days defined as accelerometer wear time ≥ 2/3 of the entire day* (Scenario 1) 9](#_Toc171350840)

[Figure 5 MAPE according to the number of valid days defined as accelerometer worn during the entire day* (Scenario 3) 10](#_Toc171350841)

[Figure 6 ICC in men according to the number of valid days defined as accelerometer wear time ≥ 2/3 of both day and night periods (Scenario 2)* 11](#_Toc171350842)

[Figure 7 ICC in women according to the number of valid days defined as accelerometer wear time ≥ 2/3 of both day and night periods (Scenario 2)* 12](#_Toc171350843)

[Figure 8 MAPE in men according to the number of valid days defined as accelerometer wear time ≥ 2/3 of both day and night periods (Scenario 2)* 13](#_Toc171350844)

[Figure 9 MAPE in women according to the number of valid days defined as accelerometer wear time ≥ 2/3 of both day and night periods (Scenario 2)* 14](#_Toc171350845)

[Figure 10 ICC in those aged <70 years according to the number of valid days defined as accelerometer wear time ≥ 2/3 of both day and night periods (Scenario 2)* 15](#_Toc171350846)

[Figure 11 MAPE in those aged <70 years according to the number of valid days defined as accelerometer wear time ≥ 2/3 of both day and night periods (Scenario 2)* 15](#_Toc171350847)

[Figure 12 ICC in those aged ≥70 years according to the number of valid days defined as accelerometer wear time ≥ 2/3 of both day and night periods (Scenario 2)* 16](#_Toc171350848)

[Figure 13 MAPE in those aged ≥70 years according to the number of valid days defined as accelerometer wear time ≥ 2/3 of both day and night periods (Scenario 2)* 18](#_Toc171350849)

[Figure 14 Illustration of the impact of imputation of a non-wear period 19](#_Toc171350850)

### Methods Rest-activity rhythm (RAR) fragmentation metrics

IS is calculated as $IS=\frac{P\sum_{h=1}^{H} {(\bar{x}_{h}-\bar{x})}^{2}}{H\sum_{p=1}^{P} {(x_{p}-\bar{x})}^{2}} , IV as IV=\frac{P\sum_{p=2}^{P} {(x_{p}-x_{p-1})}^{2}}{(P-1)\sum_{p=1}^{P} {(x_{p}-\bar{x})}^{2}} ,$where $\bar{x}_{h}$ is the h^th^ element of a vector of *H* hourly proportion of activity, $\bar{x}$ is the overall mean hourly proportion of activity over the entire period, $x_{p}$ is the p^th^ element of a vector of P hourly proportion of activity, *H* is the number of hours per day, and *P* the total number of hours over the observation period.

The TPs are calculated as follows:

- TP from activity to rest during the day (TP_ar,d_) as $TP_{ar,d}=\frac{n_{a,d}+\delta}{T_{a,d}+\delta} ,$where $n_{a,d}$is the number of bouts of activity during the day, $T_{a,d}$ is the total time of activity during the day, and $\delta$ is a small number, here it is equal to ${10}^{-6}$, to avoid potential division by zero.
- TP from wake to sleep during the night (TP_ws,n_) as $TP_{ws,n}=\frac{n_{w,n}+\delta}{T_{w,n}+\delta} ,$where $n_{w,n}$is the number of bouts of wake during the night, $T_{w,n}$ is the total waking time during the night.
- TP from rest to activity during the day (TP_ra,d_) as $TP_{ra,d}=\frac{n_{r,d}+\delta}{T_{r,d}+\delta} ,$where $n_{r,d}$is the number of bouts of rest during the day, $T_{r,d}$ is the total time of rest during the day.
- TP from sleep to wake during the night (TP_sw,n_) calculated as $TP_{sw,n}=\frac{n_{s,n}+\delta}{T_{s,n}+\delta} ,$where $n_{s,n}$is the number of bouts of sleep during the night, $T_{s,n}$ is the total time of sleep during the night.

The way to estimate α requires some intermediate steps. First, let us take the cumulative sum minus the average as $y_{t}=\sum_{i=1}^{t} (x_{i}-\bar{x}), t\leq T,$ where $y_{t}$ returns the cumulative sum of $x_{i}$ minus the average, $x_{i}$ is similar to $x_{p}$ but for any epoch length (eg, 15 seconds, 5 minutes, one hour), and *T* is the total number of epochs.

Then we organize the data in *K* nonoverlapping boxes with size equal to n as

$$b_{1}=\left( y_{1}, \ldots,y_{n} \right), b_{2}=\left( y_{n+1}, \ldots,y_{2n} \right), ..., b_{K}=\left( y_{(K-1)n+1}, \ldots,y_{Kn} \right),$$

where $Kn=T$.

Then the expected $y_{t}$is predicted for each different value of $n$ using a linear regression restricted within the $k$ box as $f_{t}\left( n \right)= \beta_{0}+ \beta_{1}t,$ where $t$ is restricted within $\left( k-1 \right)n+1 \leq t \leq kn$ and $k=1,\ldots,K$. These correspond to *K* linear regressions for each value of *n*. The root mean square fluctuation is given by $F\left( n \right)= \sqrt{\frac{1}{T}\sum_{t=1}^{T} {(y_{t}-f_{t}(n))}^{2}},$ and $\alpha$ is estimated by linear regression as $\log\left( F\left( n \right) \right)= \mu+ \alpha\log\left( n \right)+\varepsilon_{n},$ where log is the natural logarithm, $4\leq n\leq T/4,$and $\varepsilon_{n}$ is a Gaussian error.

ABI is a transformation of $\alpha$, which is obtained as $ABI={exp}^{{-|\alpha-1|}/{{exp}^{-2}}} ,$ where *exp* is the natural exponential.

### Methods to compare results from pseudo-simulation

For each i^th^ individual we have a reference value for each of the eight RAR fragmentation metrics (RAR_i,ref_) described in the section above using his/her complete signal over the full seven days. For each RAR fragmentation metric, each i^th^ individual has a simulated value of RAR fragmentation metric (RAR_i,sim, b_) for each one of the 20 simulated batches. To evaluate the performance of the b^th^ simulation we use the summary metric intraclass correlation coefficient (ICC), which is calculated as

$$ICC\left( RAR,b \right)=\frac{1}{n\sigma^{2}}\sum_{i=1}^{n} ({RAR}_{i,sim,b}-\mu)({RAR}_{i,ref}-\mu),$$

where $\mu$ is the mean of all individuals in ${RAR}_{i,sim,b}$ and ${RAR}_{i,ref}$ , $\sigma^{2}$ is the variance of all individuals in ${RAR}_{i,sim,b}$ and ${RAR}_{i,ref}$ .

The mean absolute percent error (MAPE) is calculated as

$$MAPE\left( RAR,b \right)=\frac{100}{n}\sum_{i=1}^{n} \left| \frac{{RAR}_{i,ref}-{RAR}_{i,sim,b}}{{RAR}_{i,ref}} \right|.$$

| **Table 1** Sample characteristics by selection criteria in the Whitehall II accelerometer substudy | | | |
| --- | --- | --- | --- |
|  | **N = 1241**  At least one entire day with accelerometer wear time≥ 2/3 of the entire day*, but accelerometer not worn full time during seven days* | **N = 2859**  Accelerometer worn during seven entire days* | **p-value** |
| Age (years), M (SD) | 69.90 (5.82) | 69.16 (5.65) | <0.001 |
| Men | 778 (62.7%) | 2257 (78.9%) | <0.001 |
| White ethnicity | 1147 (92.5%) | 2638 (92.3%) | 0.914 |
| Education |  |  | 0.097 |
| No academic qualifications | 132 (10.6%) | 250 (8.7%) |  |
| Lower secondary school | 387 (31.2%) | 920 (32.2%) |  |
| Higher secondary school | 329 (26.5%) | 810 (28.3%) |  |
| University | 303(24.4%) | 639 (22.4%) |  |
| Higher degree | 89 (7.2%) | 240 (8.4%) |  |
| Not married/cohabitating | 390 (31.5%) | 649 (22.7%) | <0.001 |
| Body mass index | 26.33 (4.39) | 26.72 (4.30) | 0.008 |

**Notes:** Data are N (%), otherwise specified. The p-value is estimated using Student’s t-test for continuous variable and chi-square test for counting data.

**Abbreviations:** mean (M), standard deviation (SD).

* An entire day is defined as the period between wake up to next wake up (it combines the day (wake up to sleep onset) and night (sleep onset to wake up) periods).

| Figure 1 Study flowchart … |
| --- |
| 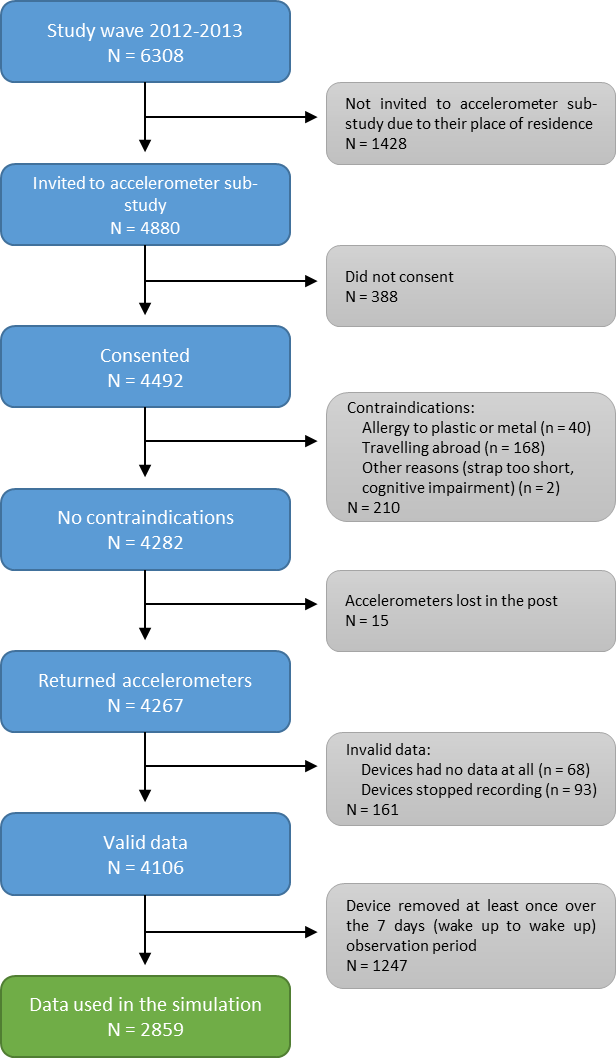 |

| **Figure 3** ICC according to the number of valid days defined as accelerometer worn during the entire day* (Scenario 3) | |
| --- | --- |
| 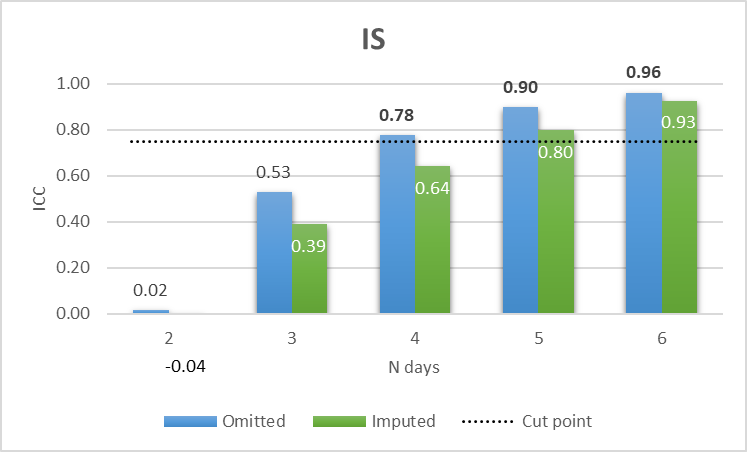 | 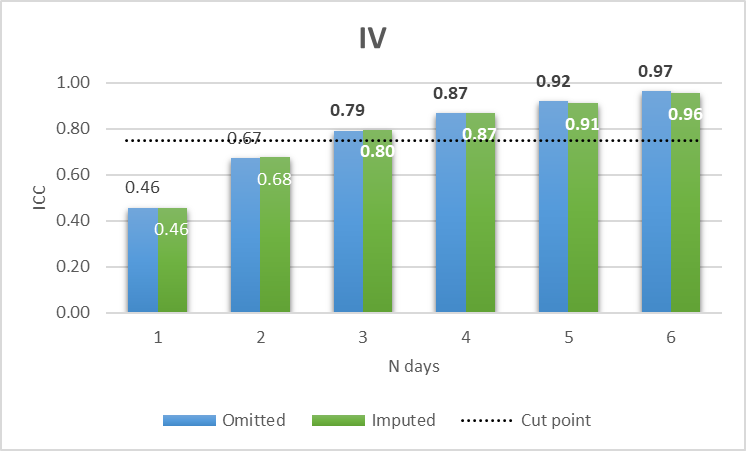 |
| 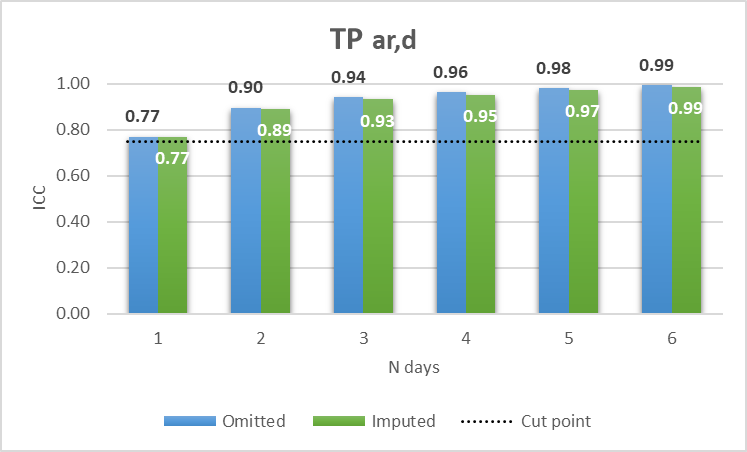 | 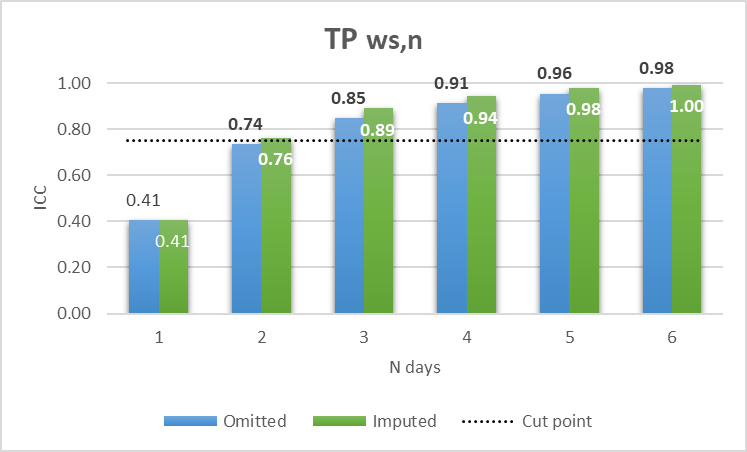 |
| 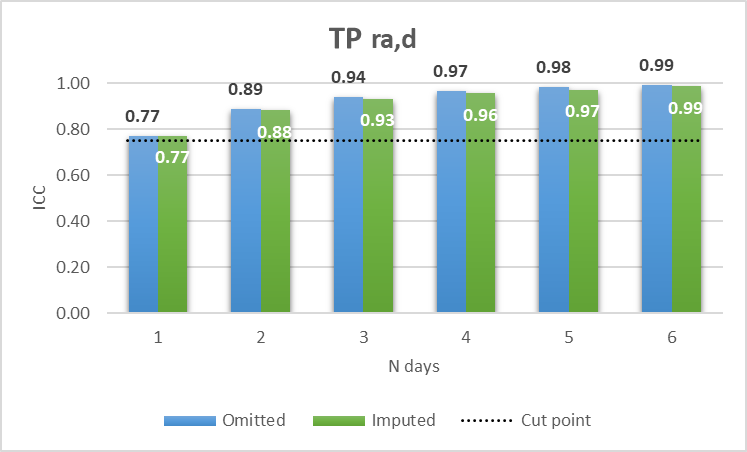 | 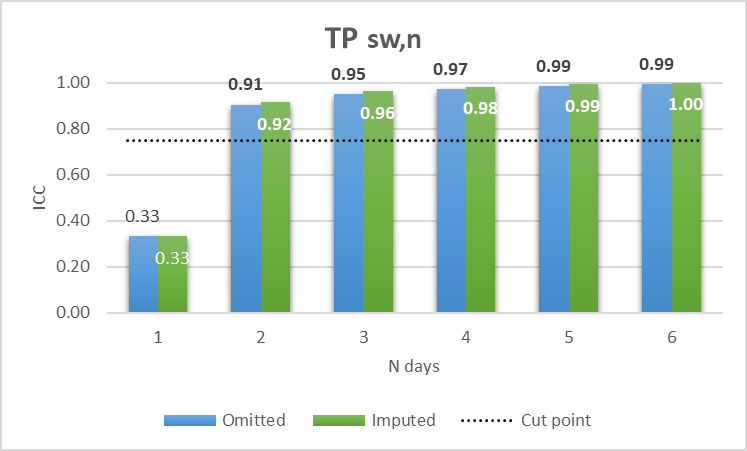 |
| 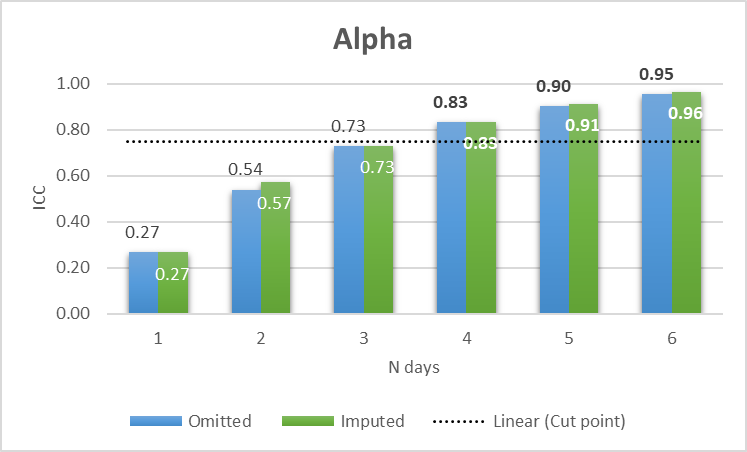 | 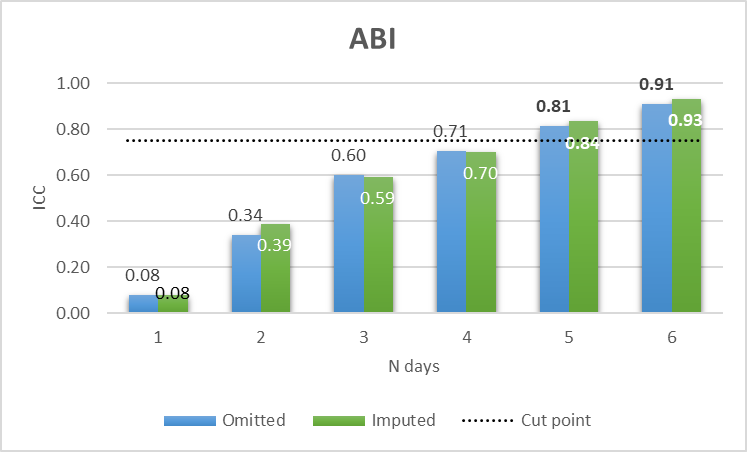 |
| **Abbreviations:** intraclass correlation coefficient (ICC), inter-daily stability (IS), intradaily variability (IV), transition probability (TP), TP from activity to rest during the day (TP_ar,d_), TP from wake to sleep during the night (TP_ws,n_), TP from rest to activity during the day (TP_ra,d_), TP from sleep to wake during the night (TP_sw,n_), and activity balance index (ABI).  Bold values correspond to ICC≥0.75  * An entire day is defined as the period between wake up to next wake up (it combines the day (wake up to sleep onset) and night (sleep onset to wake up) periods). | |
| **Figure 4** MAPE according to the number of valid days defined as accelerometer wear time ≥ 2/3 of the entire day* (Scenario 1) | |
| 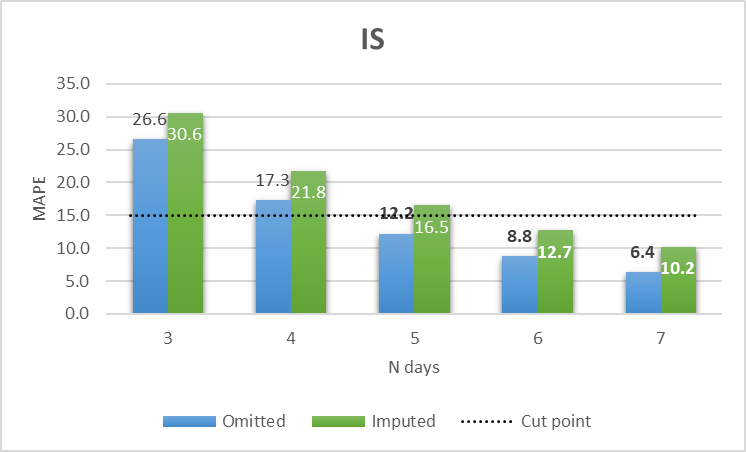 | 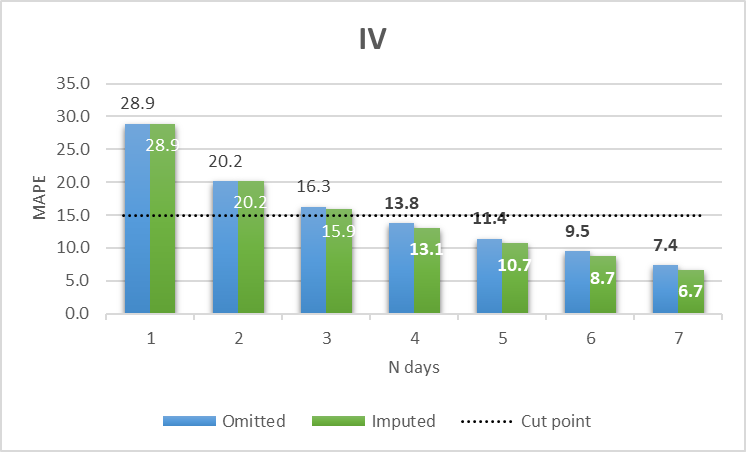 |
| 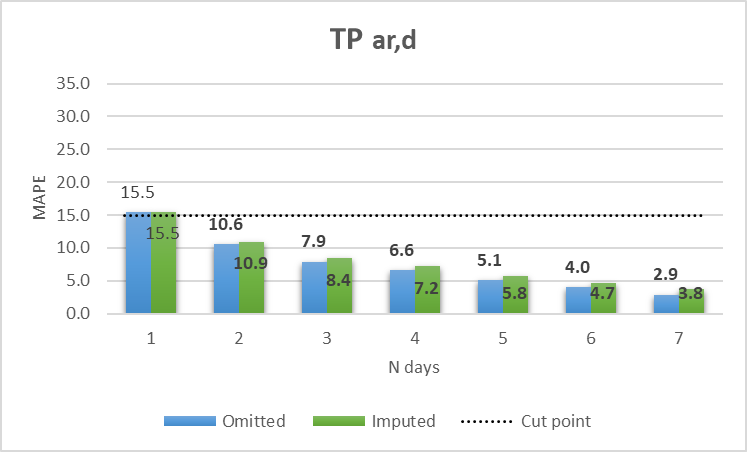 | 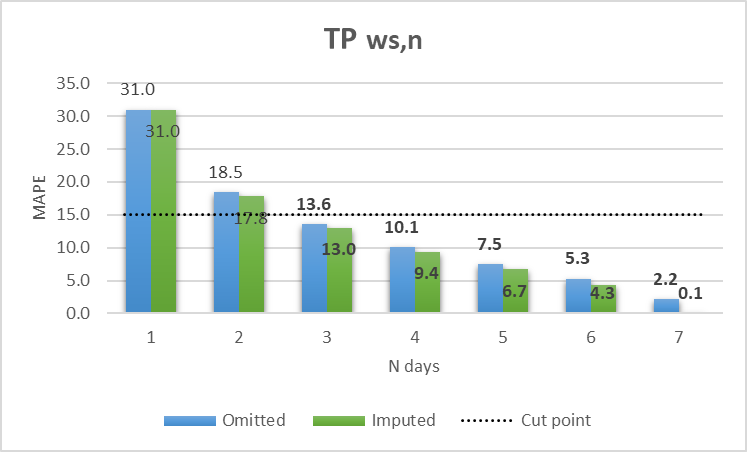 |
| 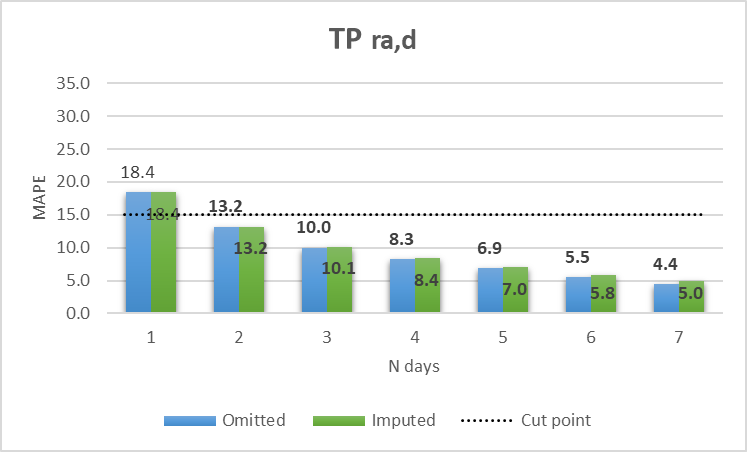 | 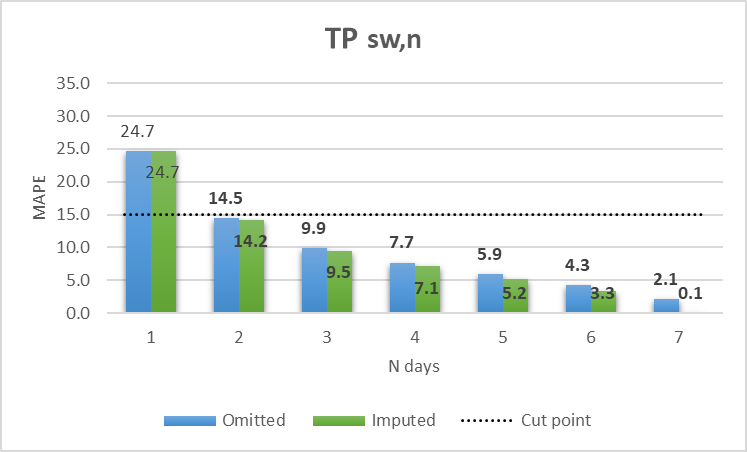 |
| 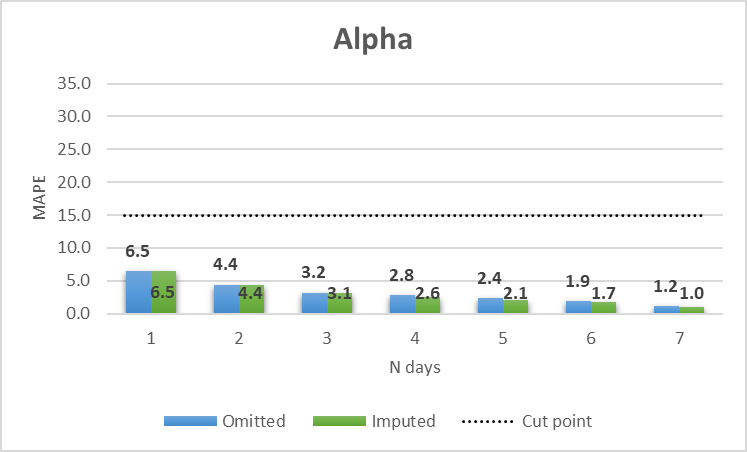 | 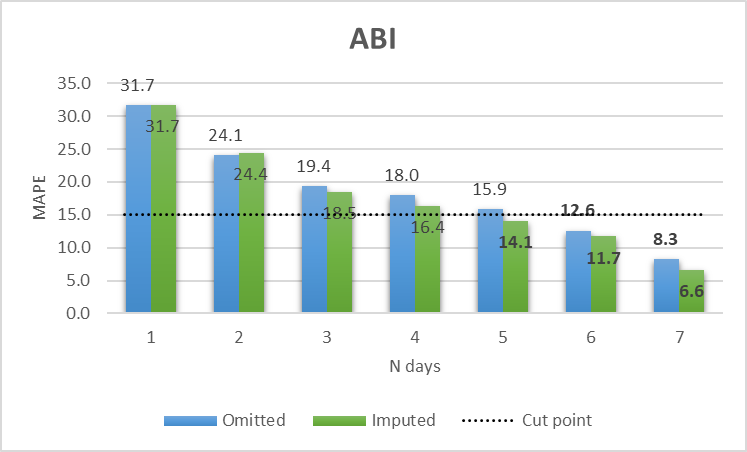 |
| **Abbreviations:** mean absolute percent error (MAPE), inter-daily stability (IS), intradaily variability (IV), transition probability (TP), TP from activity to rest during the day (TP_ar,d_), TP from wake to sleep during the night (TP_ws,n_), TP from rest to activity during the day (TP_ra,d_), TP from sleep to wake during the night (TP_sw,n_), and activity balance index (ABI).  Bold values correspond to MAPE≤15%  * An entire day is defined as the period between wake up to next wake up (it combines the day (wake up to sleep onset) and night (sleep onset to wake up) periods). | |

| **Figure 5** MAPE according to the number of valid days defined as accelerometer worn during the entire day* (Scenario 3) | |
| --- | --- |
| 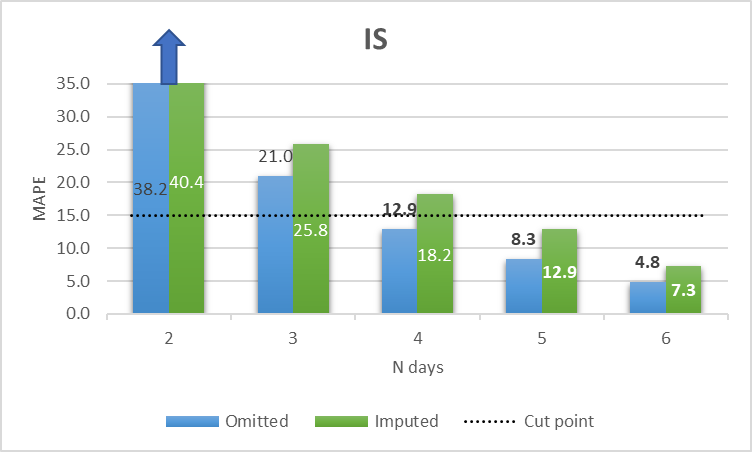 | 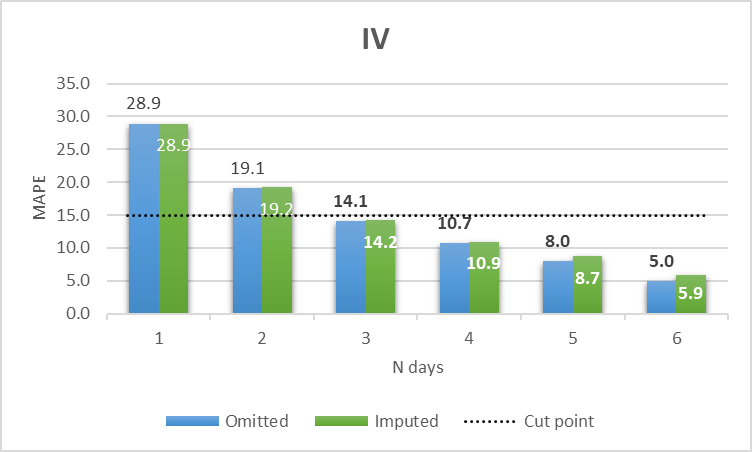 |
| 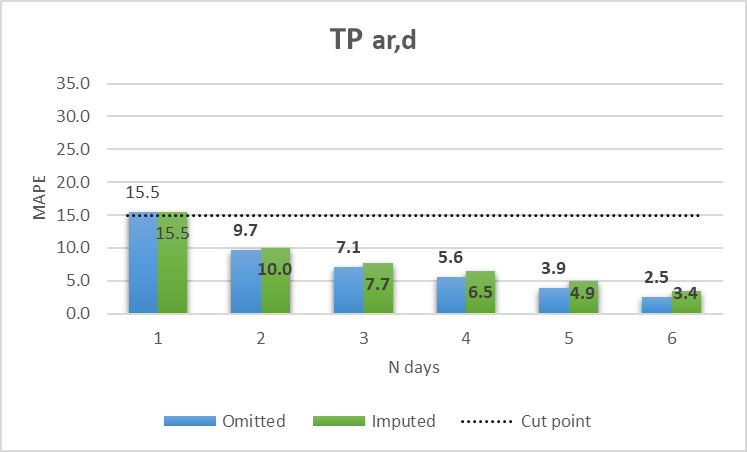 | 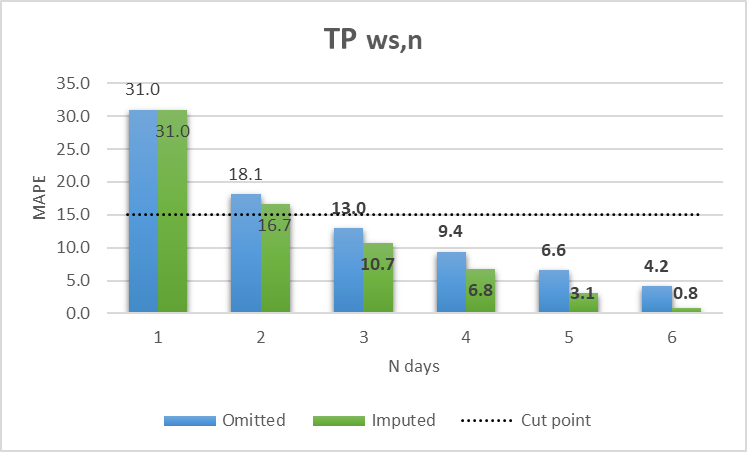 |
| 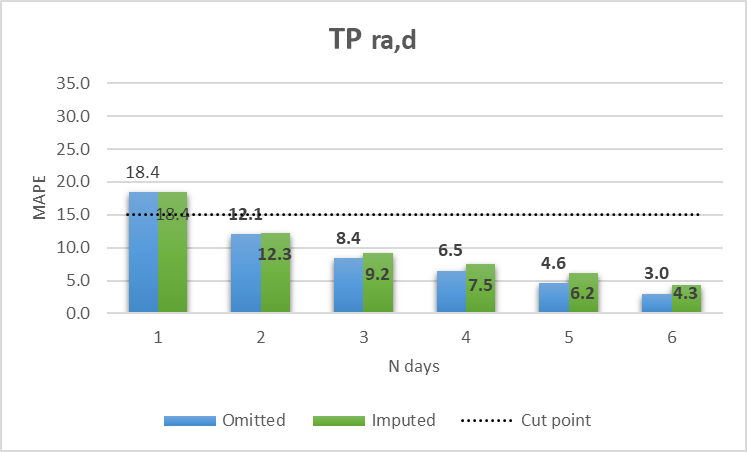 | 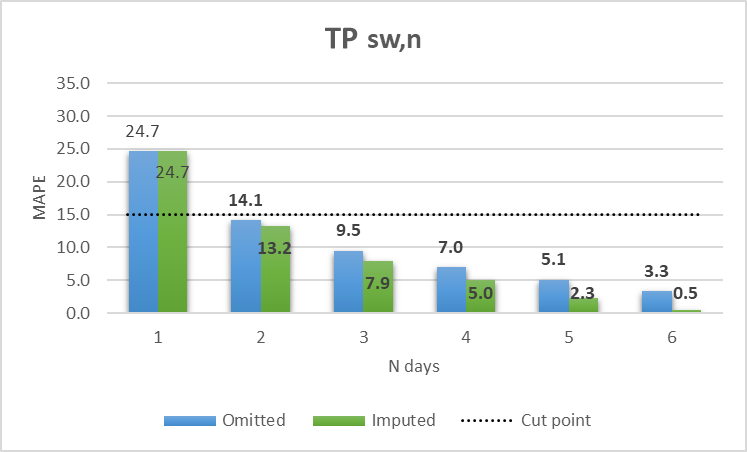 |
| 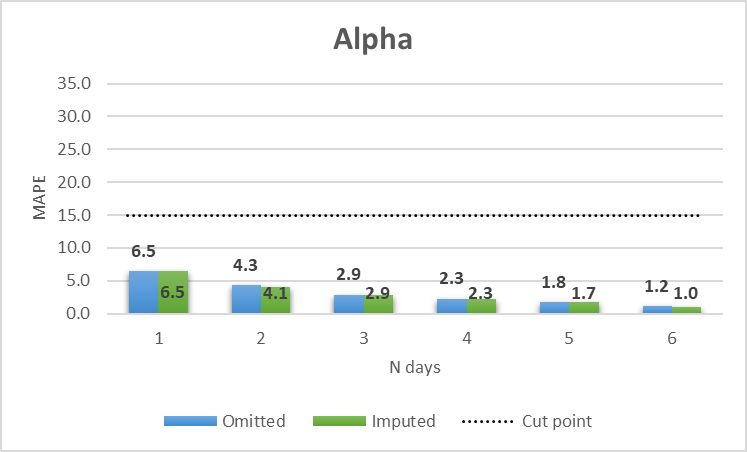 | 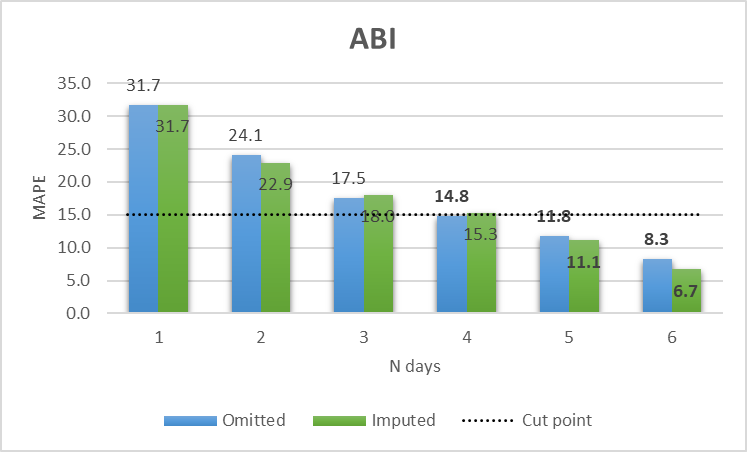 |
| **Abbreviations:** mean absolute percent error (MAPE), inter-daily stability (IS), intradaily variability (IV), transition probability (TP), TP from activity to rest during the day (TP_ar,d_), TP from wake to sleep during the night (TP_ws,n_), TP from rest to activity during the day (TP_ra,d_), TP from sleep to wake during the night (TP_sw,n_), and activity balance index (ABI).  Bold values correspond to MAPE≤15%  * An entire day is defined as the period between wake up to next wake up (it combines the day (wake up to sleep onset) and night (sleep onset to wake up) periods). | |
| \| **Figure 6** ICC in men according to the number of valid days defined as accelerometer wear time ≥ 2/3 of both day and night periods (Scenario 2)* \| \| \| --- \| --- \| \| 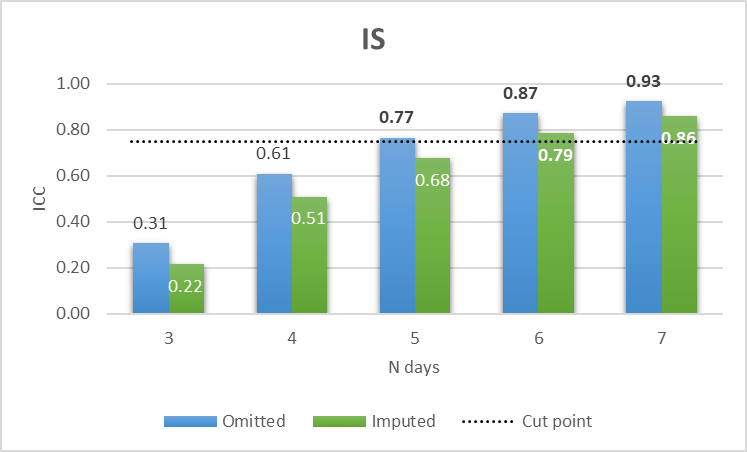 \| 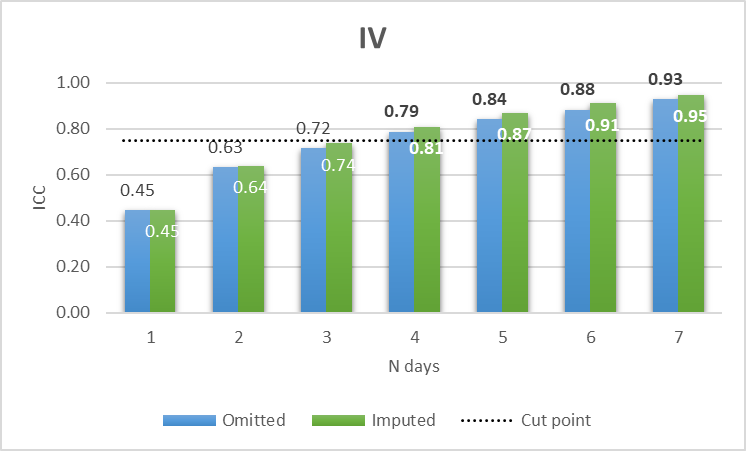 \| \| 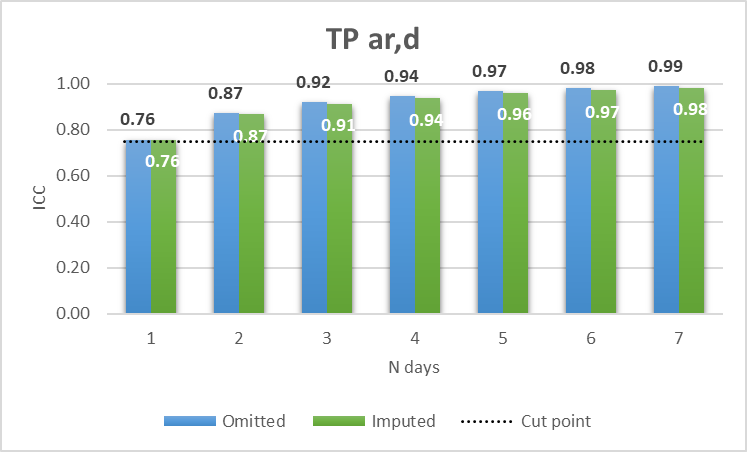 \| 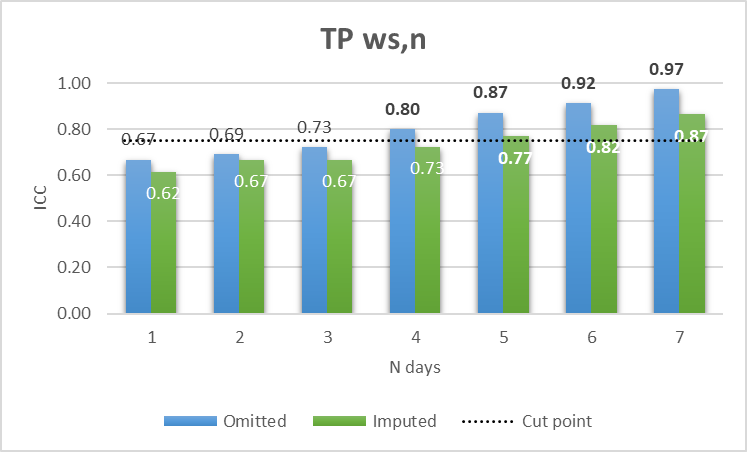 \| \| 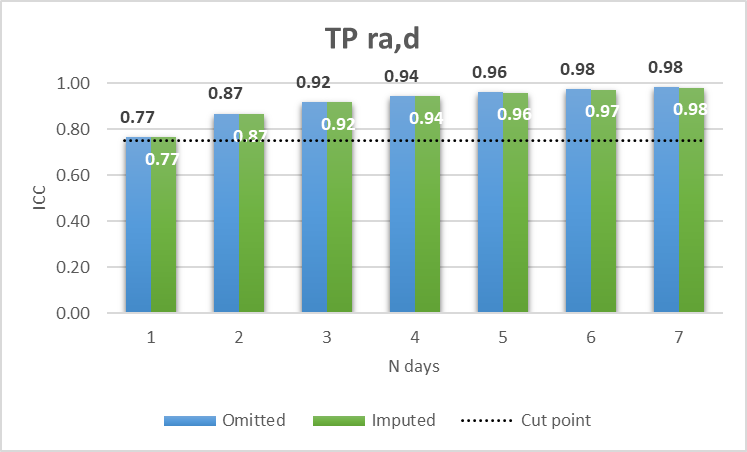 \| 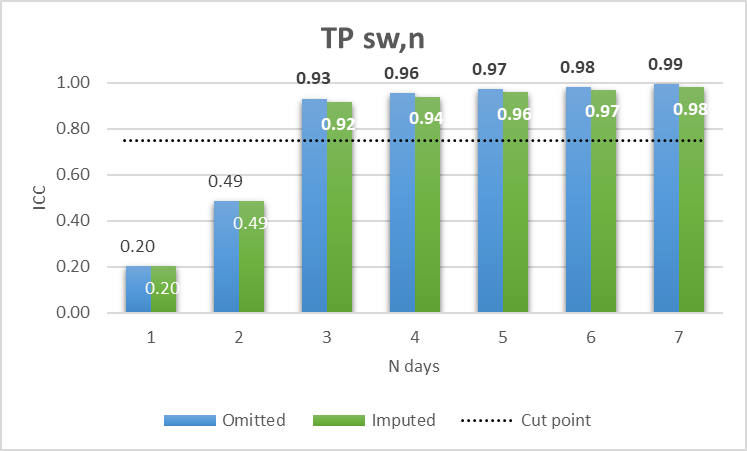 \| \| 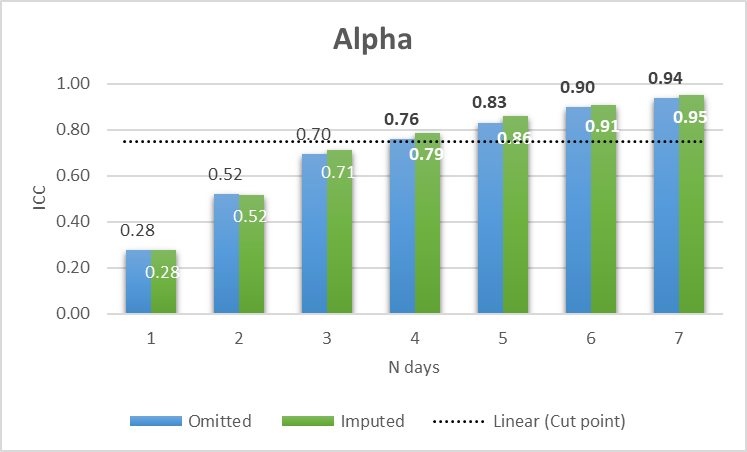 \| 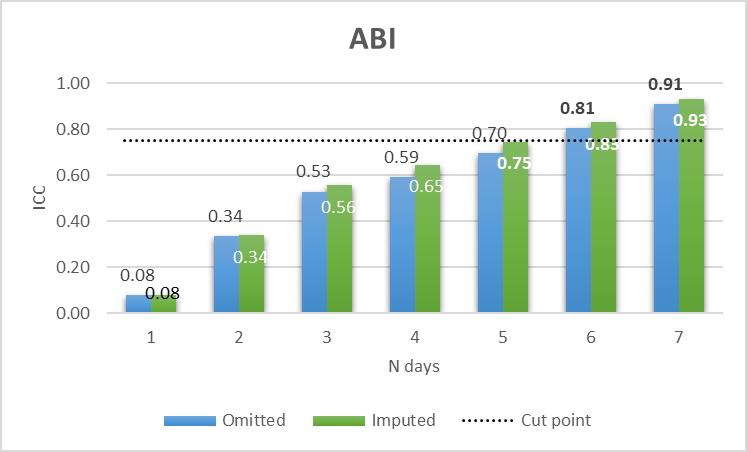 \| \| **Abbreviations:** intraclass correlation coefficient (ICC), inter-daily stability (IS), intradaily variability (IV), transition probability (TP), TP from activity to rest during the day (TP_ar,d_), TP from wake to sleep during the night (TP_ws,n_), TP from rest to activity during the day (TP_ra,d_), TP from sleep to wake during the night (TP_sw,n_), and activity balance index (ABI).  Bold values correspond to ICC≥0.75  * An entire day is defined as the period between wake up to next wake up (it combines the day (wake up to sleep onset) and night (sleep onset to wake up) periods). \| \|  \| **Figure 7** ICC in women according to the number of valid days defined as accelerometer wear time ≥ 2/3 of both day and night periods (Scenario 2)* \| \| \| --- \| --- \| \| 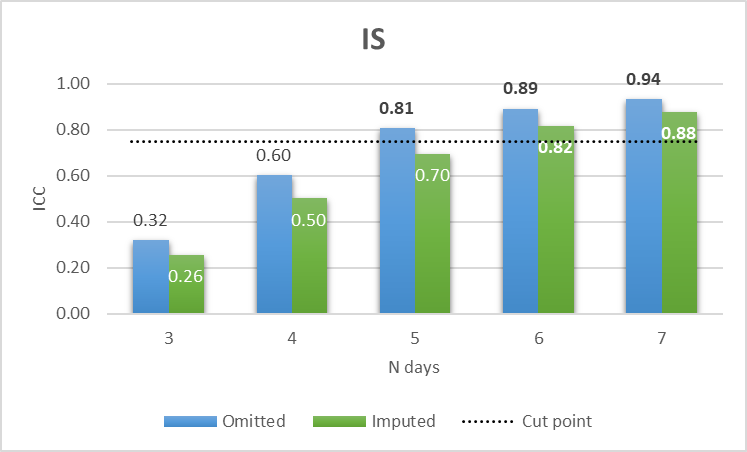 \| 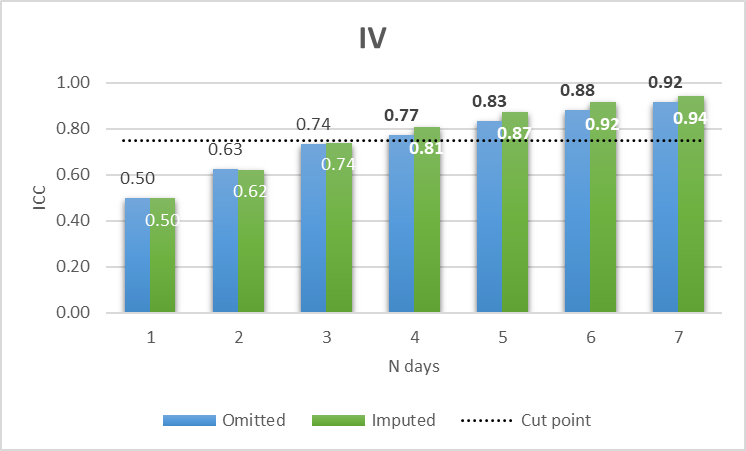 \| \| 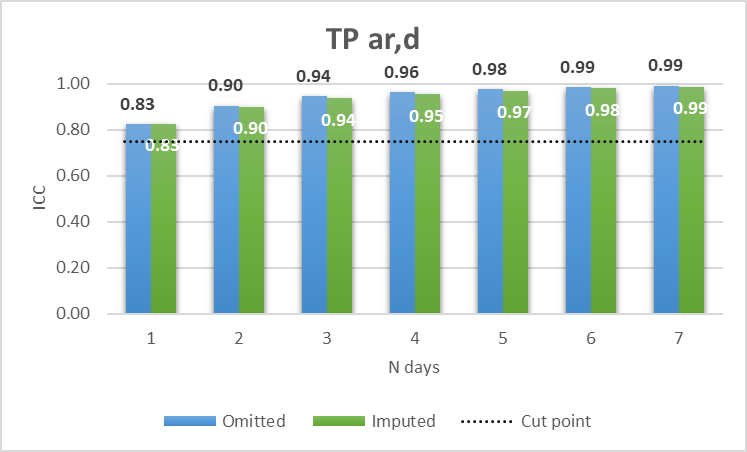 \| 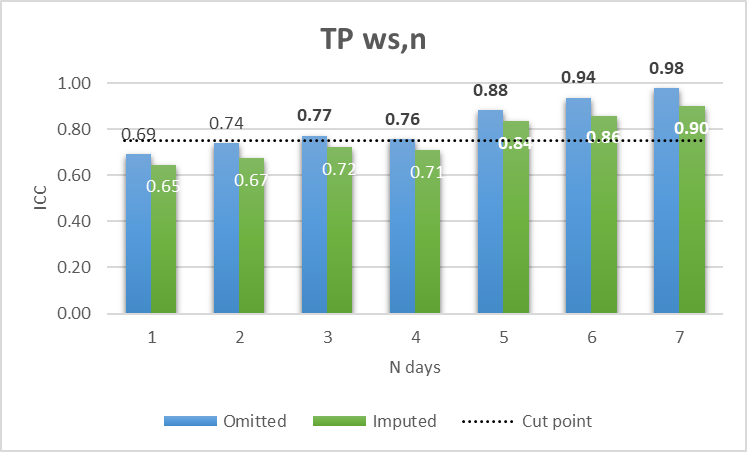 \| \| 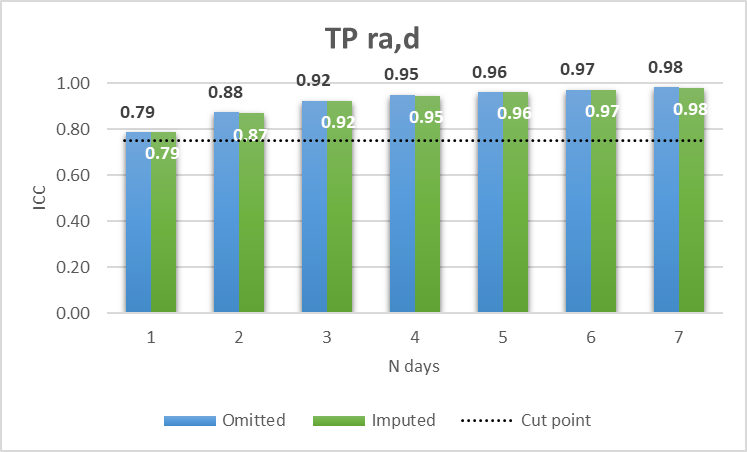 \| 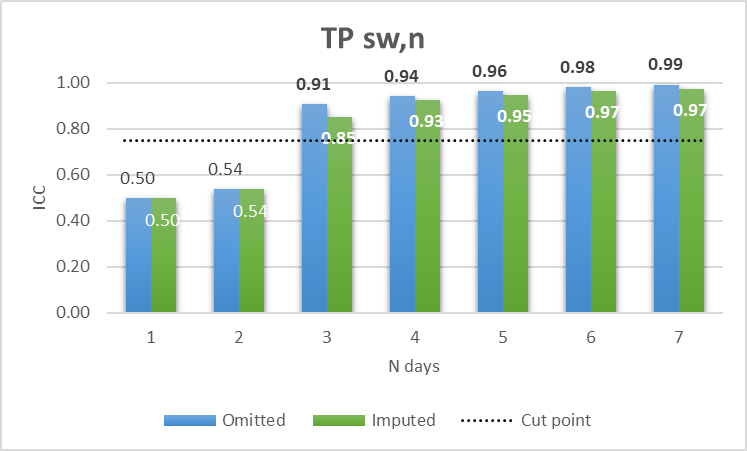 \| \| 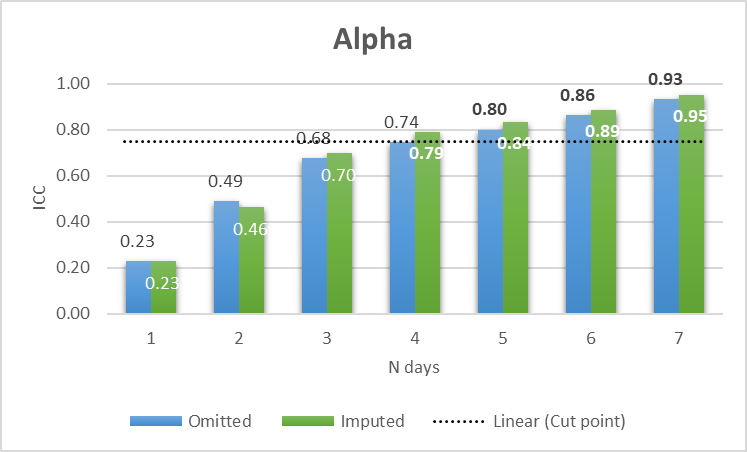 \| 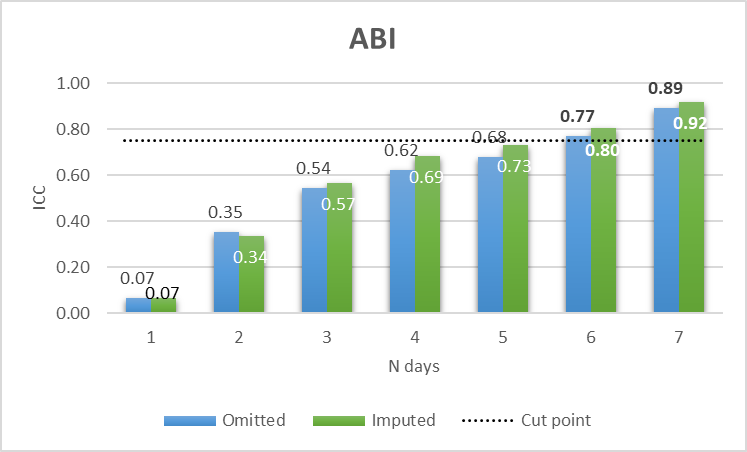 \| \| **Abbreviations:** intraclass correlation coefficient (ICC), inter-daily stability (IS), intradaily variability (IV), transition probability (TP), TP from activity to rest during the day (TP_ar,d_), TP from wake to sleep during the night (TP_ws,n_), TP from rest to activity during the day (TP_ra,d_), TP from sleep to wake during the night (TP_sw,n_), and activity balance index (ABI).  Bold values correspond to ICC≥0.75  * An entire day is defined as the period between wake up to next wake up (it combines the day (wake up to sleep onset) and night (sleep onset to wake up) periods). \| \| | |
| \| **Figure 8** MAPE in men according to the number of valid days defined as accelerometer wear time ≥ 2/3 of both day and night periods (Scenario 2)* \| \| \| --- \| --- \| \| 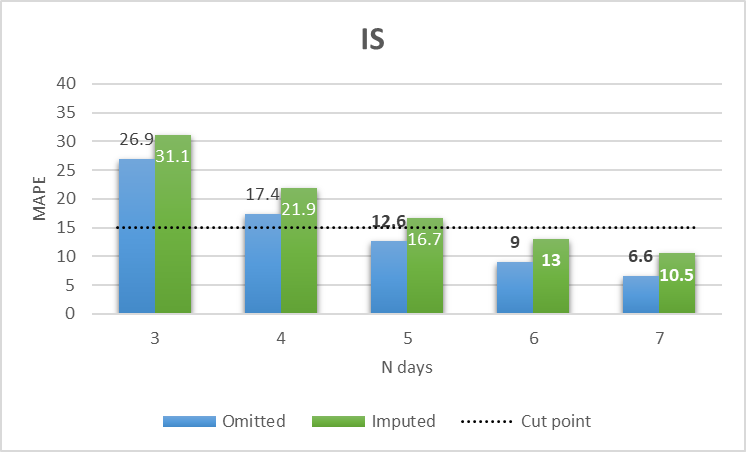 \| 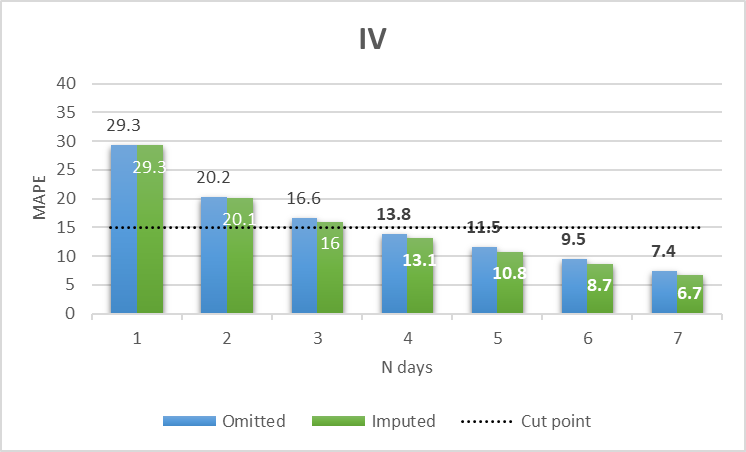 \| \| 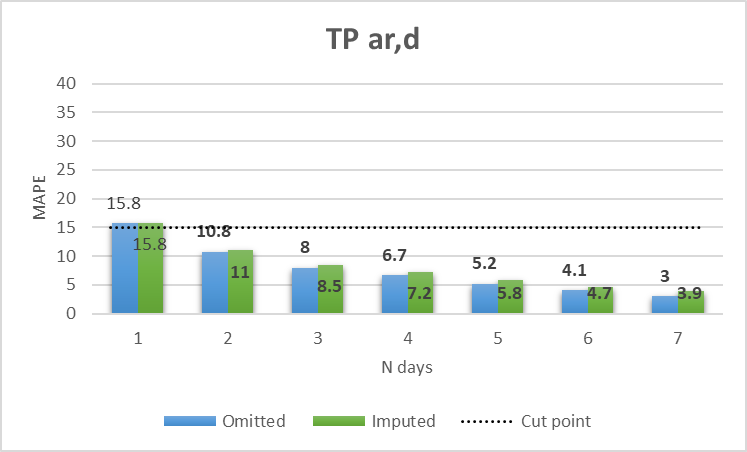 \| 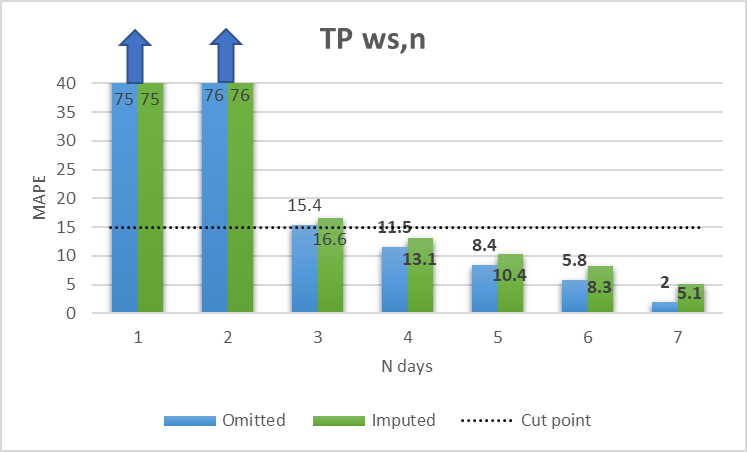 \| \| 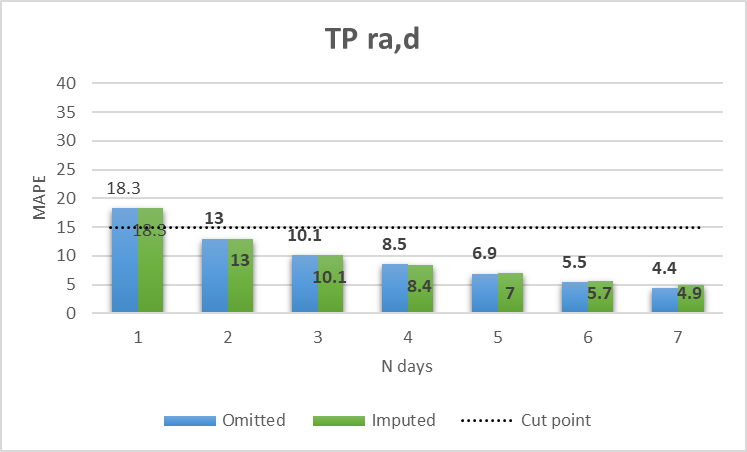 \| 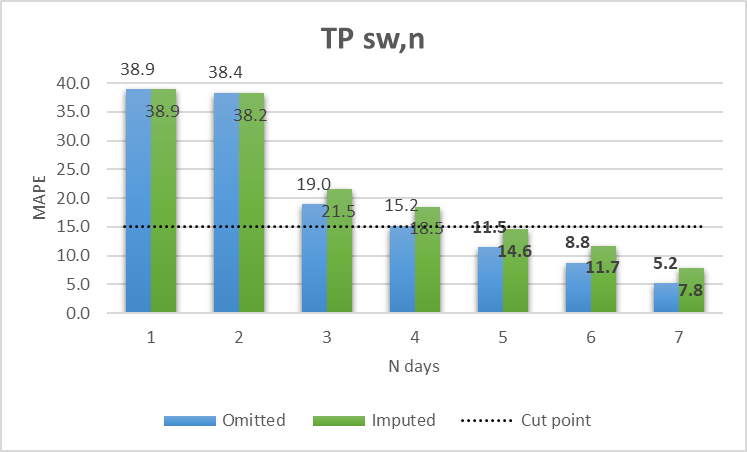 \| \| 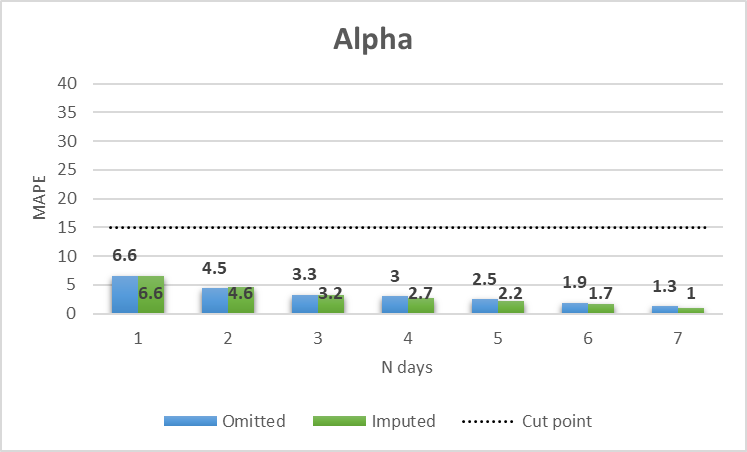 \| 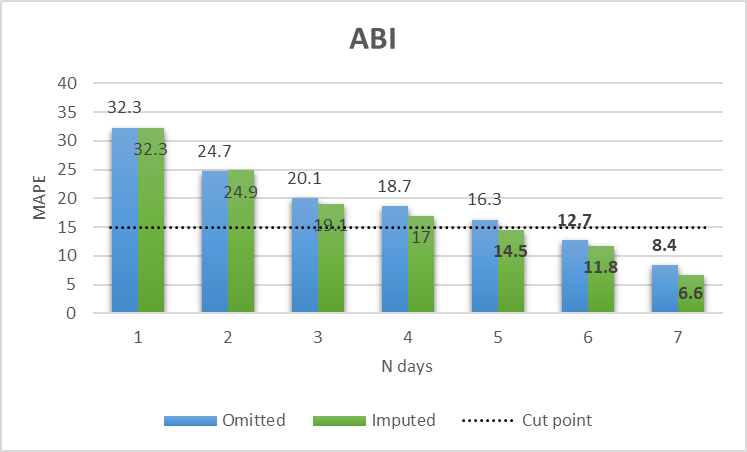 \| \| **Abbreviations:** mean absolute percent error (MAPE), inter-daily stability (IS), intradaily variability (IV), transition probability (TP), TP from activity to rest during the day (TP_ar,d_), TP from wake to sleep during the night (TP_ws,n_), TP from rest to activity during the day (TP_ra,d_), TP from sleep to wake during the night (TP_sw,n_), and activity balance index (ABI).  Bold values correspond to MAPE≤15%  * An entire day is defined as the period between wake up to next wake up (it combines the day (wake up to sleep onset) and night (sleep onset to wake up) periods). \| \|  \| **Figure 9** MAPE in women according to the number of valid days defined as accelerometer wear time ≥ 2/3 of both day and night periods (Scenario 2)* \| \| \| --- \| --- \| \| 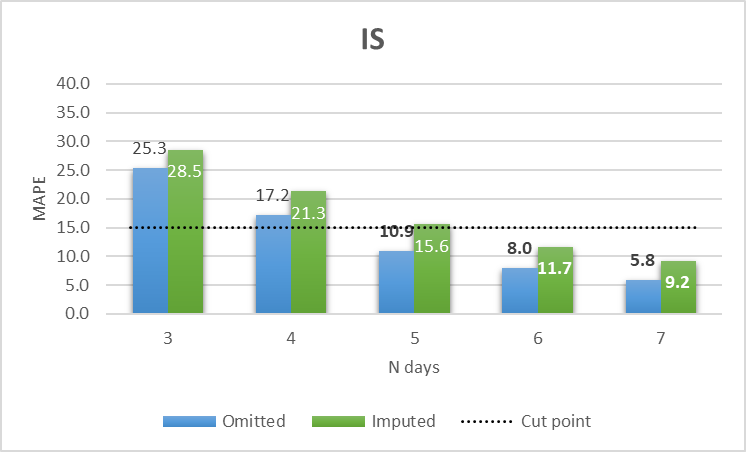 \| 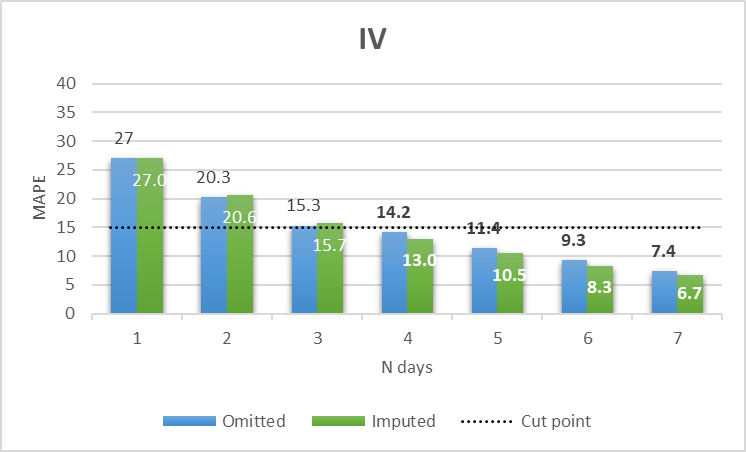 \| \| 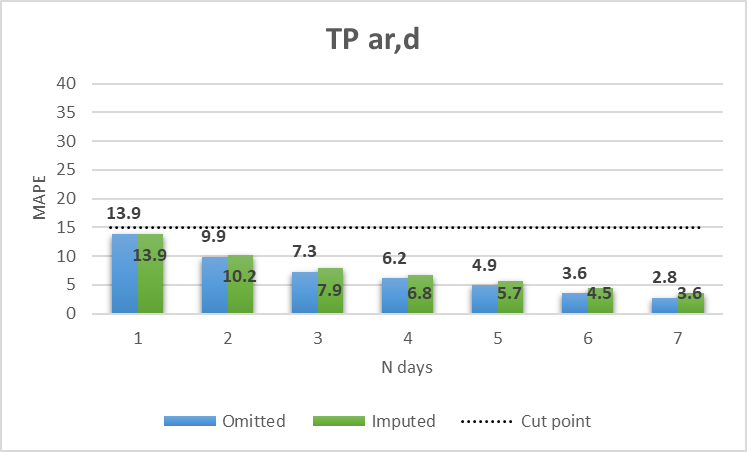 \| 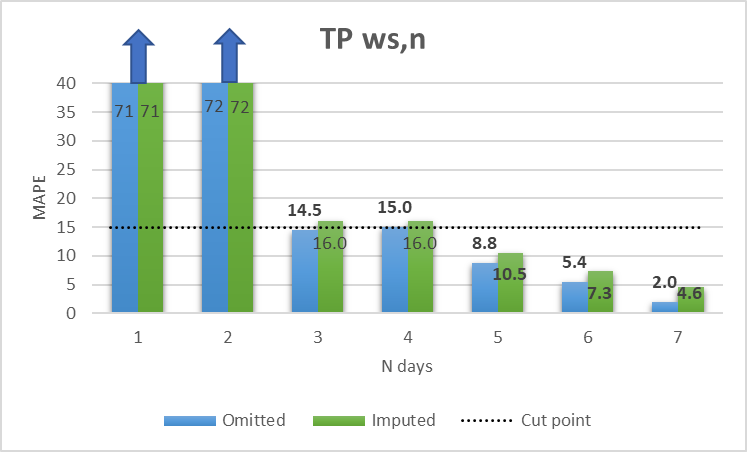 \| \| 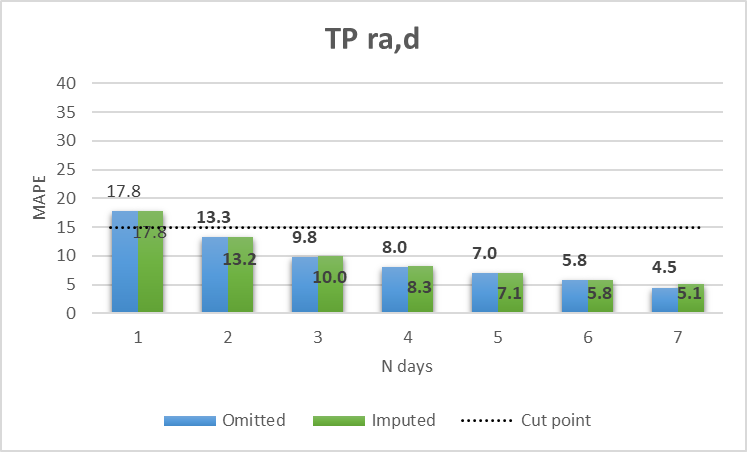 \| 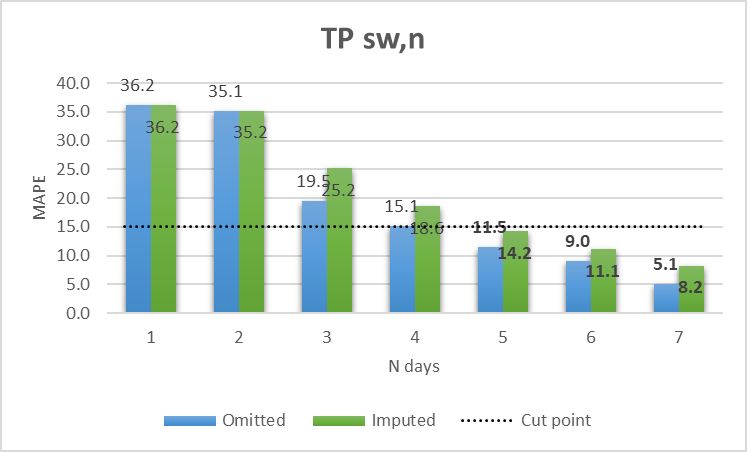 \| \| 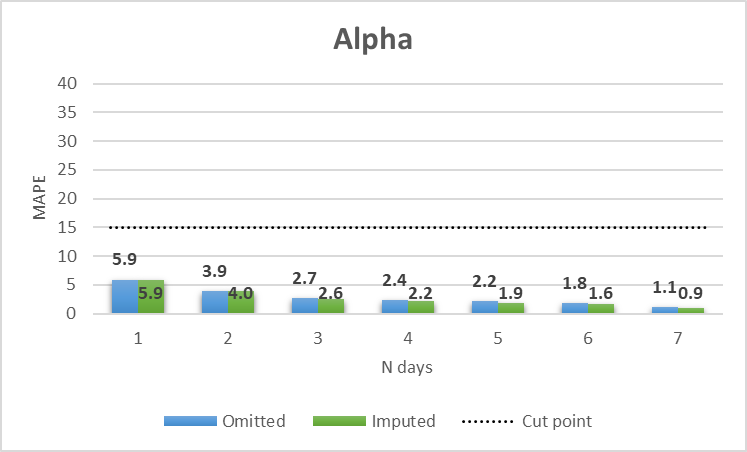 \| 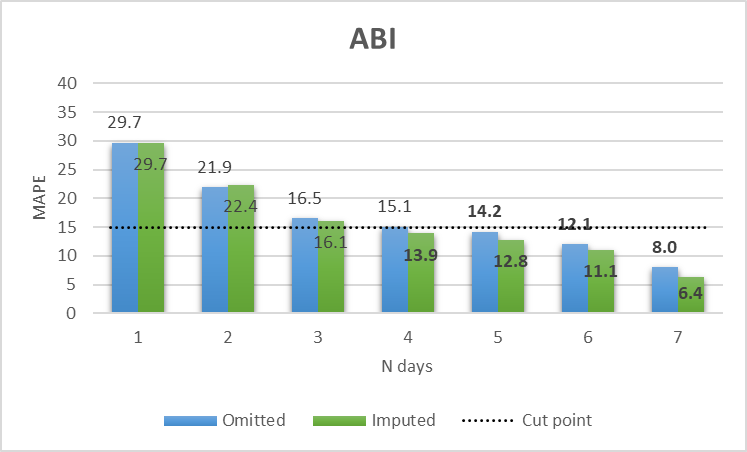 \| \| **Abbreviations:** mean absolute percent error (MAPE), inter-daily stability (IS), intradaily variability (IV), transition probability (TP), TP from activity to rest during the day (TP_ar,d_), TP from wake to sleep during the night (TP_ws,n_), TP from rest to activity during the day (TP_ra,d_), TP from sleep to wake during the night (TP_sw,n_), and activity balance index (ABI).  Bold values correspond to MAPE≤15%  * An entire day is defined as the period between wake up to next wake up (it combines the day (wake up to sleep onset) and night (sleep onset to wake up) periods). \| \|  \| **Figure 10** ICC in those aged <70 years according to the number of valid days defined as accelerometer wear time ≥ 2/3 of both day and night periods (Scenario 2)* \| \| \| --- \| --- \| \| 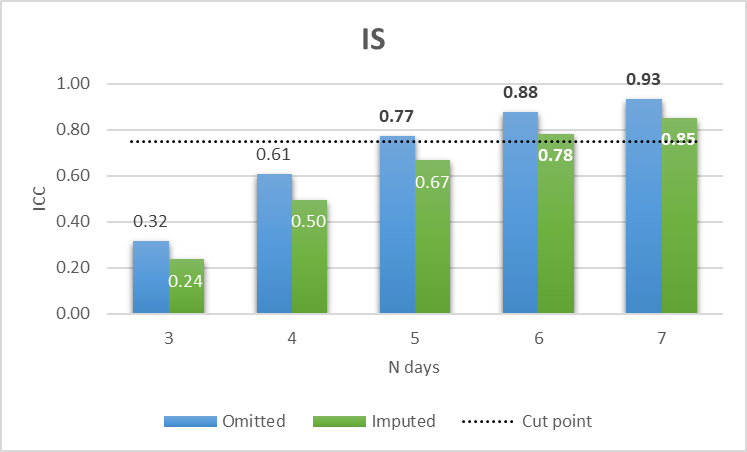 \| 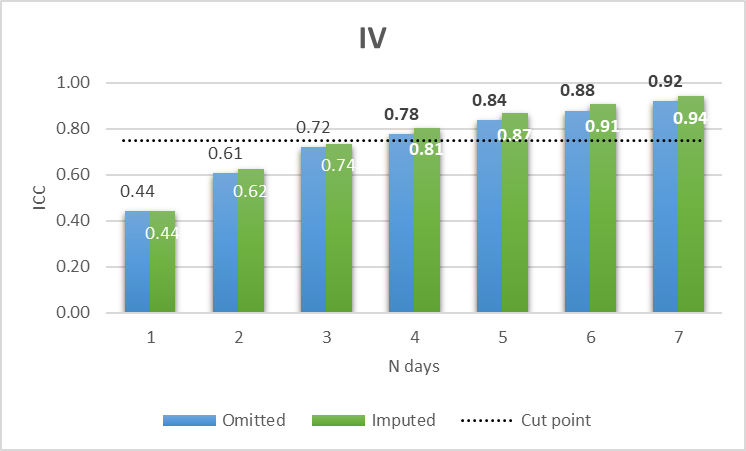 \| \| 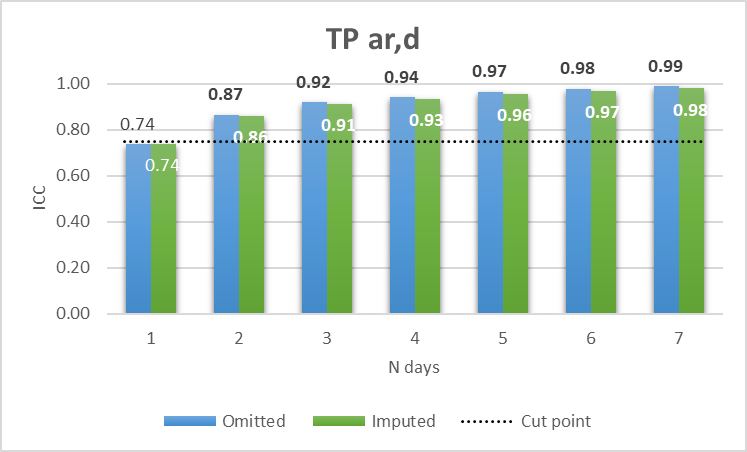 \| 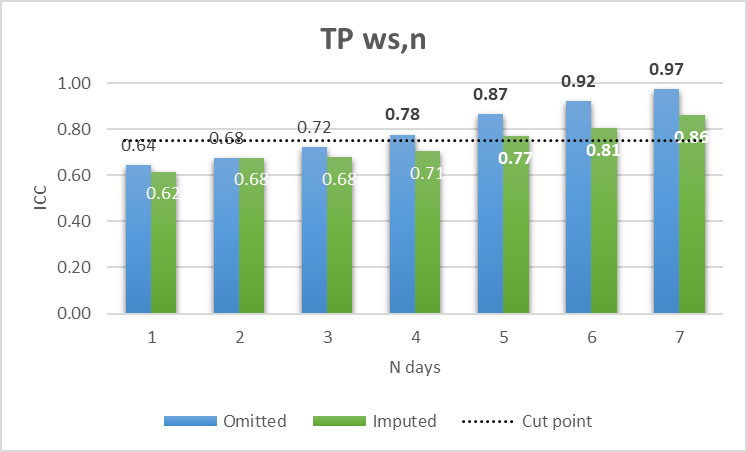 \| \| 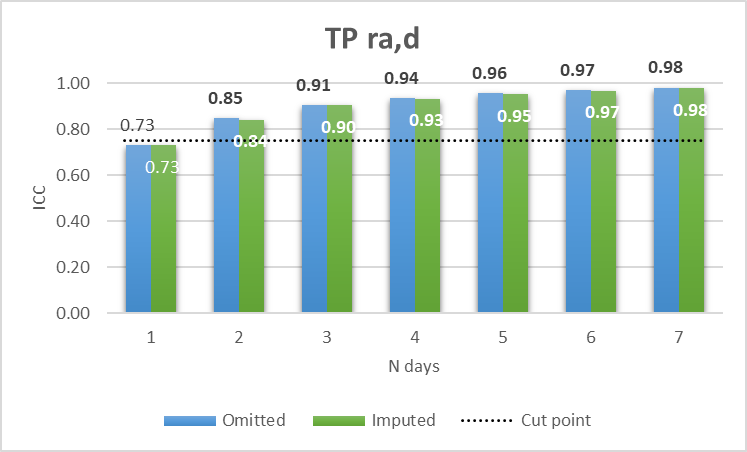 \| 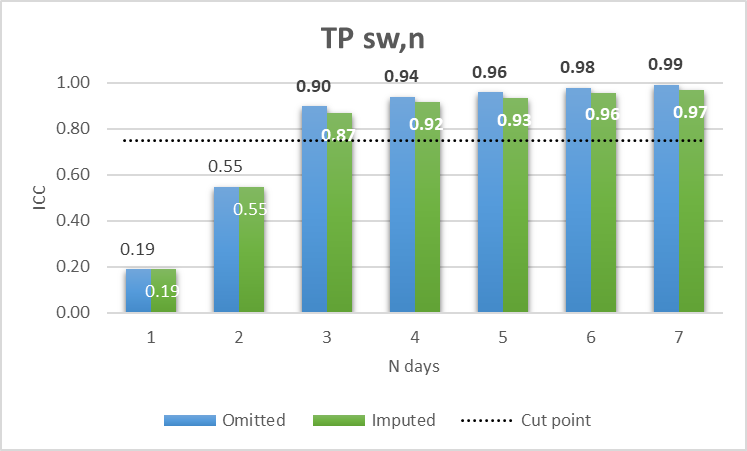 \| \| 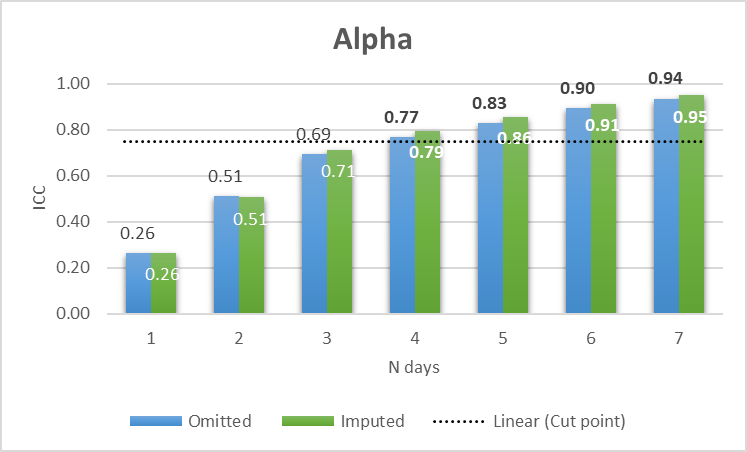 \| 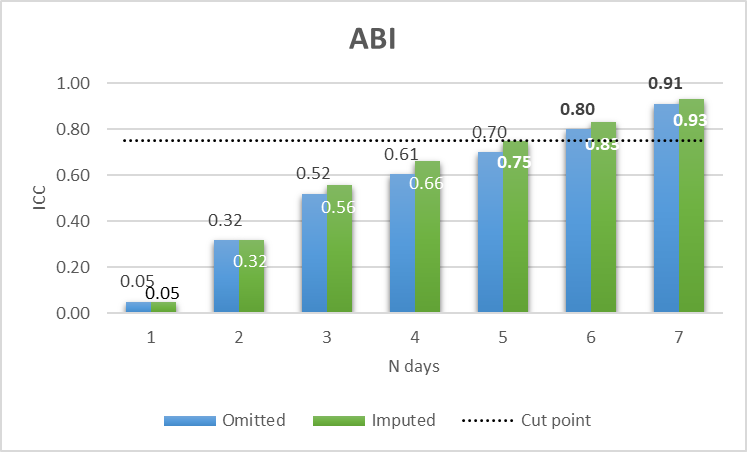 \| \| **Abbreviations:** intraclass correlation coefficient (ICC), inter-daily stability (IS), intradaily variability (IV), transition probability (TP), TP from activity to rest during the day (TP_ar,d_), TP from wake to sleep during the night (TP_ws,n_), TP from rest to activity during the day (TP_ra,d_), TP from sleep to wake during the night (TP_sw,n_), and activity balance index (ABI).  Bold values correspond to ICC≥0.75  * An entire day is defined as the period between wake up to next wake up (it combines the day (wake up to sleep onset) and night (sleep onset to wake up) periods). \| \|  \| **Figure 11** MAPE in those aged <70 years according to the number of valid days defined as accelerometer wear time ≥ 2/3 of both day and night periods (Scenario 2)* \| \| \| --- \| --- \| \| 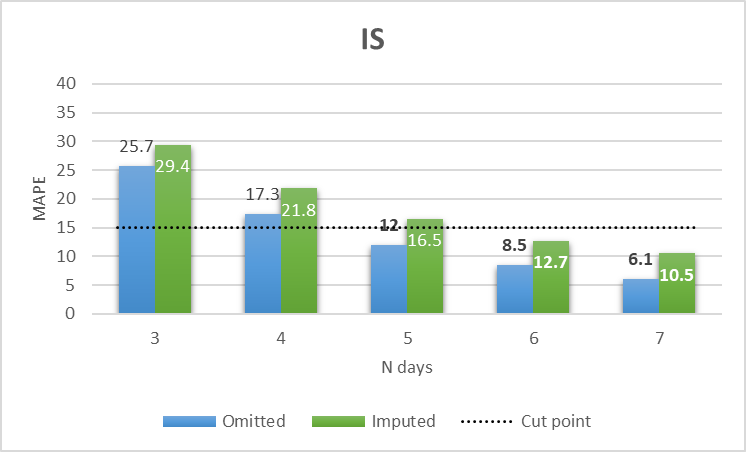 \| 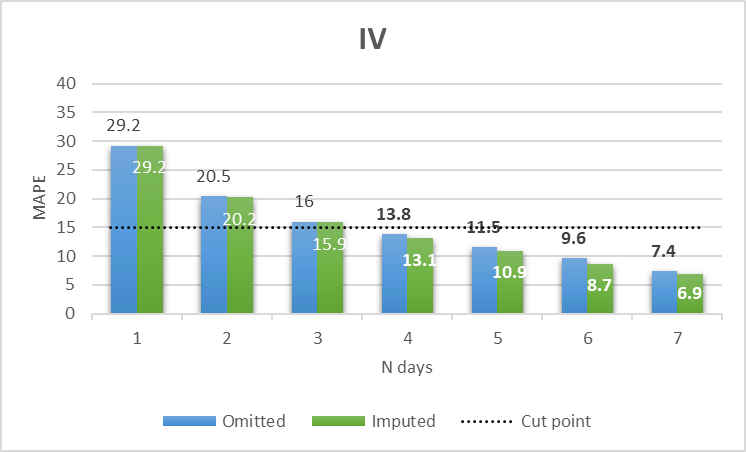 \| \| 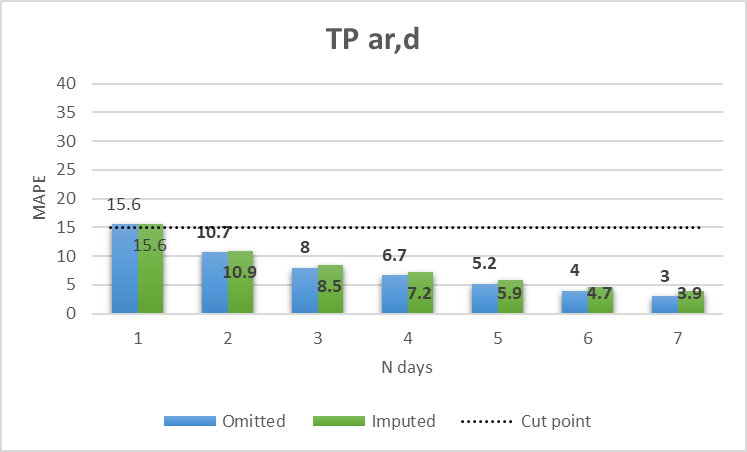 \| 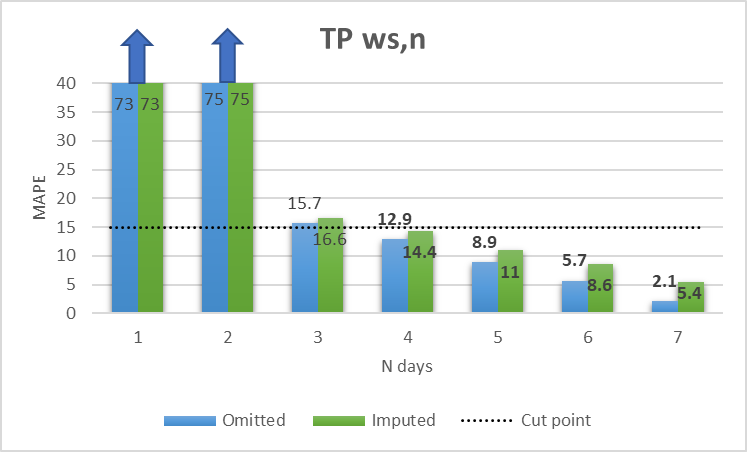 \| \| 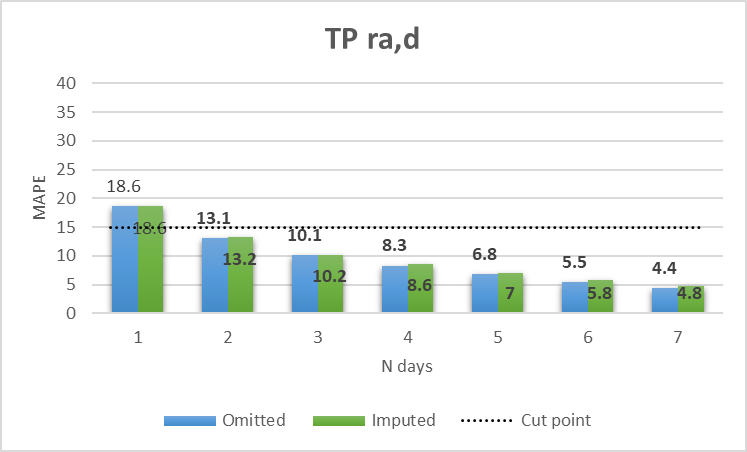 \| 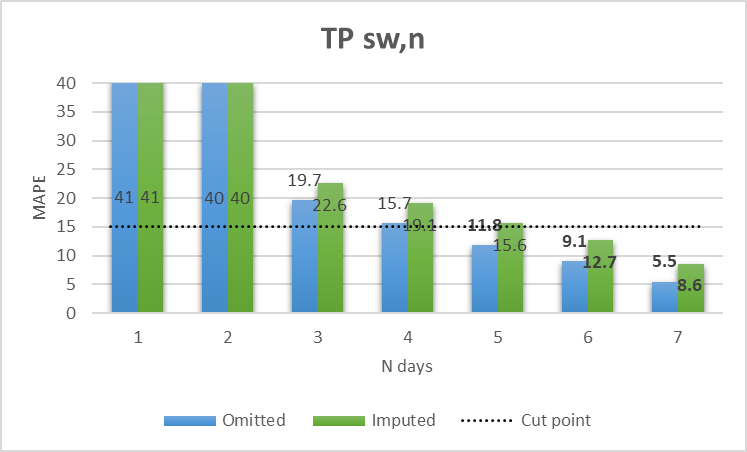 \| \| 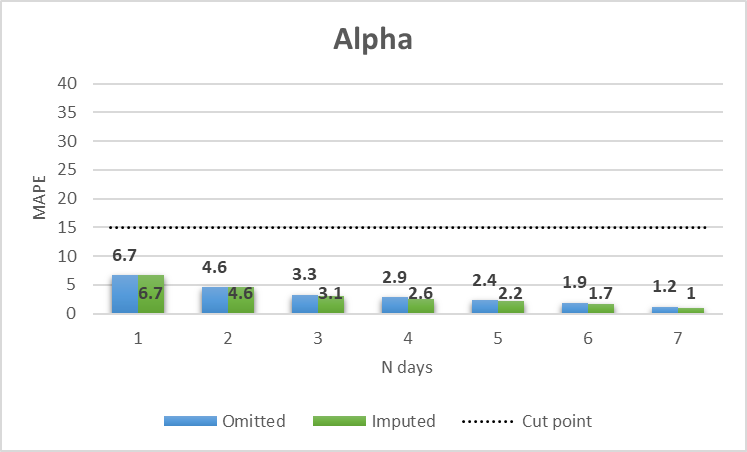 \| 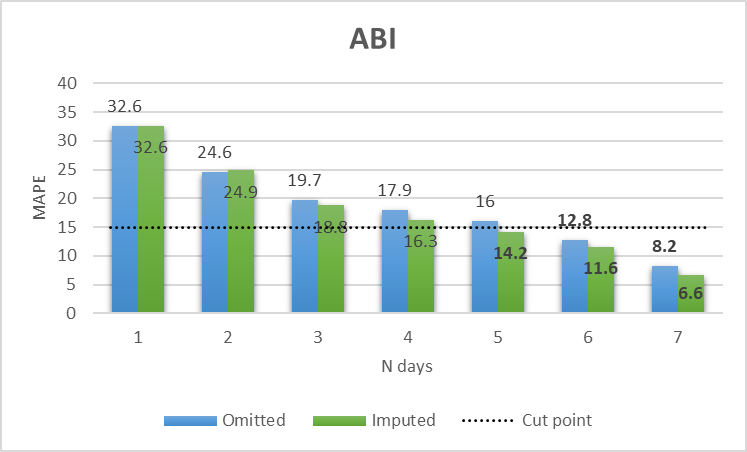 \| \| **Abbreviations:** mean absolute percent error (MAPE), inter-daily stability (IS), intradaily variability (IV), transition probability (TP), TP from activity to rest during the day (TP_ar,d_), TP from wake to sleep during the night (TP_ws,n_), TP from rest to activity during the day (TP_ra,d_), TP from sleep to wake during the night (TP_sw,n_), and activity balance index (ABI).  Bold values correspond to MAPE≤15%  * An entire day is defined as the period between wake up to next wake up (it combines the day (wake up to sleep onset) and night (sleep onset to wake up) periods). \| \|  **Figure 12** ICC in those aged ≥70 years according to the number of valid days defined as accelerometer wear time ≥ 2/3 of both day and night periods (Scenario 2)* | |
| 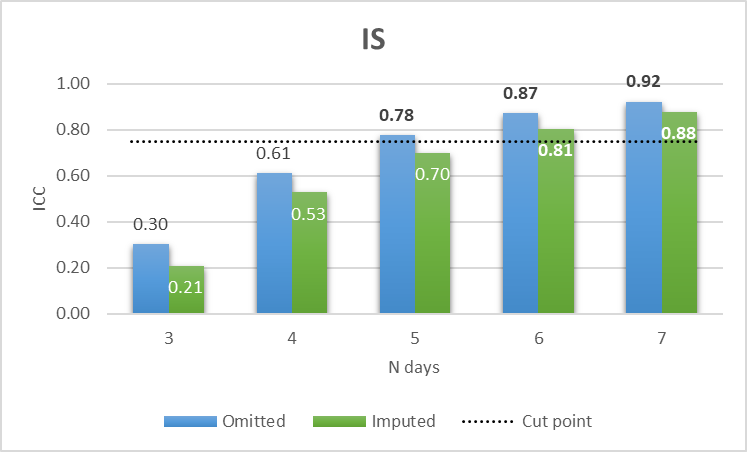 | 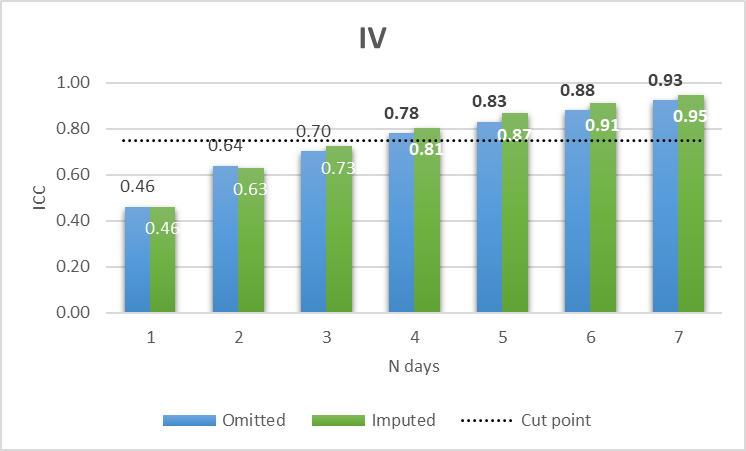 |
| 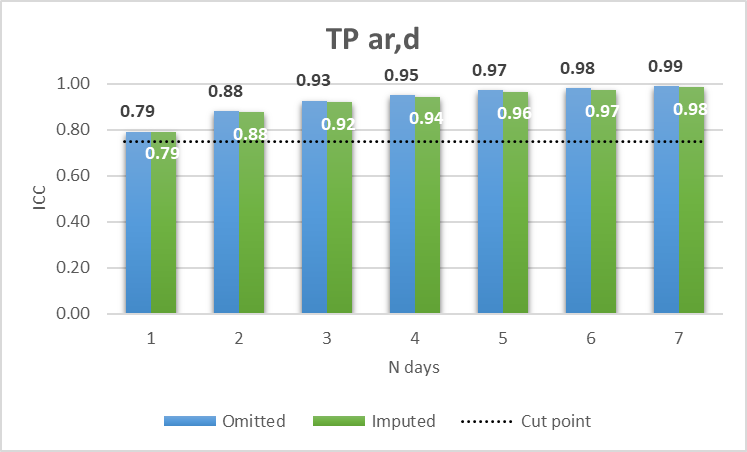 | 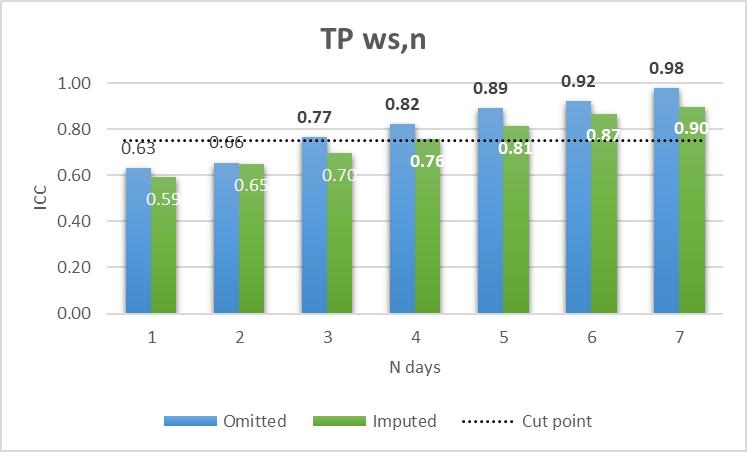 |
| 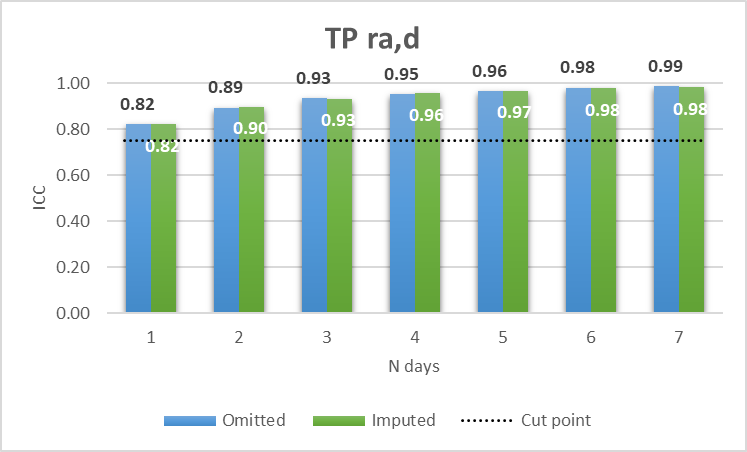 | 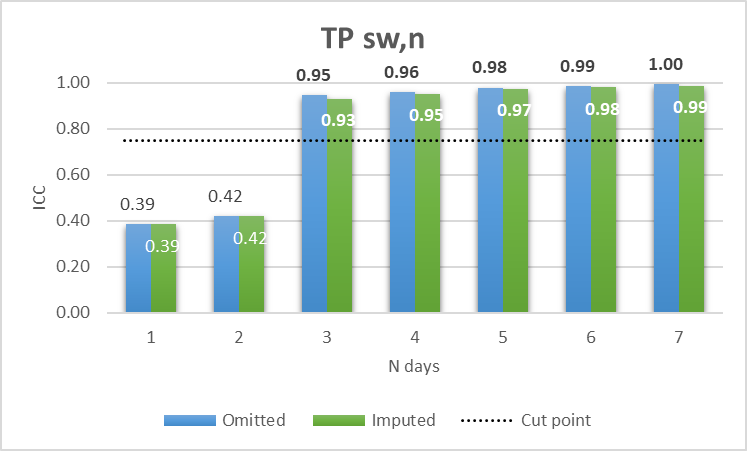 |
| 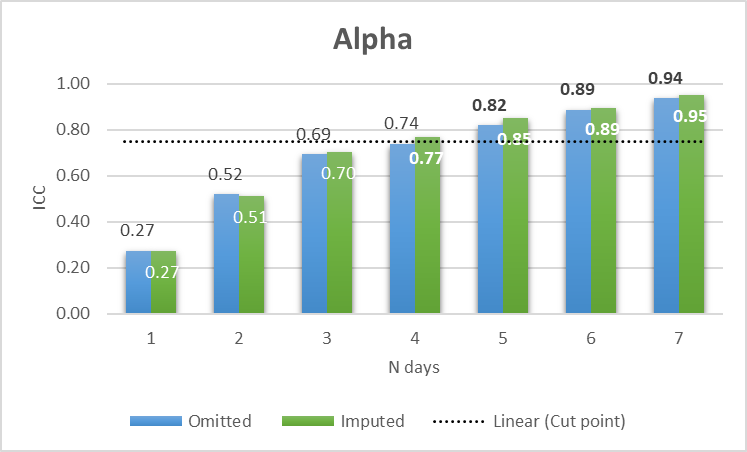 | 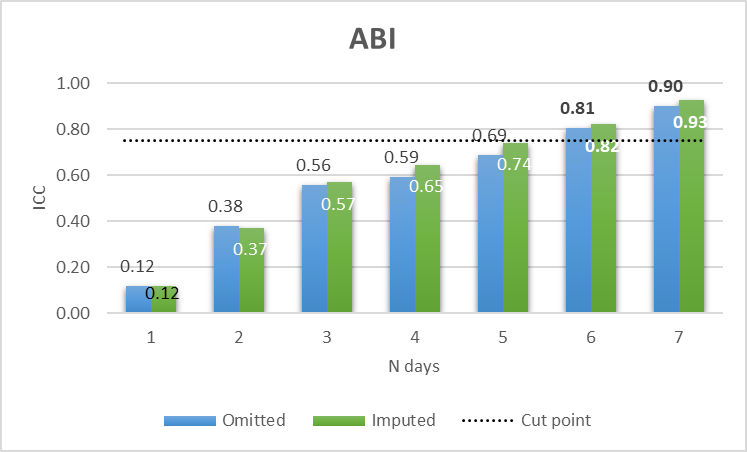 |
| **Abbreviations:** intraclass correlation coefficient (ICC), inter-daily stability (IS), intradaily variability (IV), transition probability (TP), TP from activity to rest during the day (TP_ar,d_), TP from wake to sleep during the night (TP_ws,n_), TP from rest to activity during the day (TP_ra,d_), TP from sleep to wake during the night (TP_sw,n_), and activity balance index (ABI).  Bold values correspond to ICC≥0.75  * An entire day is defined as the period between wake up to next wake up (it combines the day (wake up to sleep onset) and night (sleep onset to wake up) periods). | |

| **Figure 13** MAPE in those aged ≥70 years according to the number of valid days defined as accelerometer wear time ≥ 2/3 of both day and night periods (Scenario 2)* | |
| --- | --- |
| 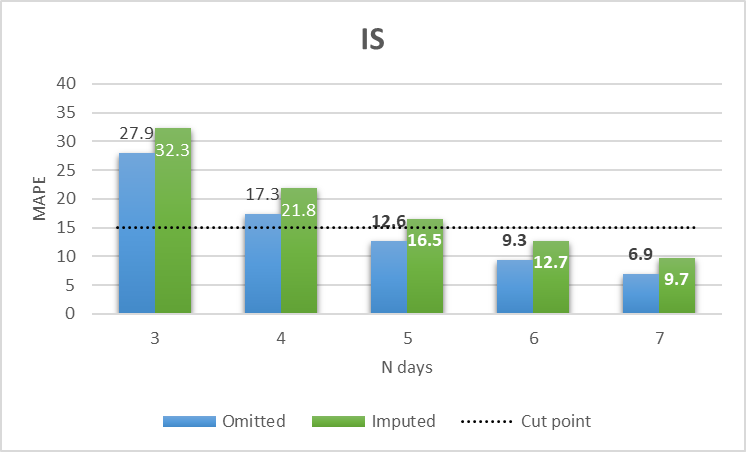 | 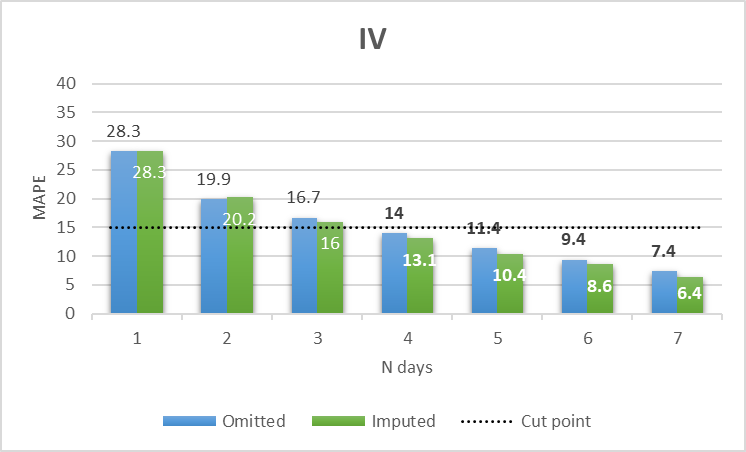 |
| 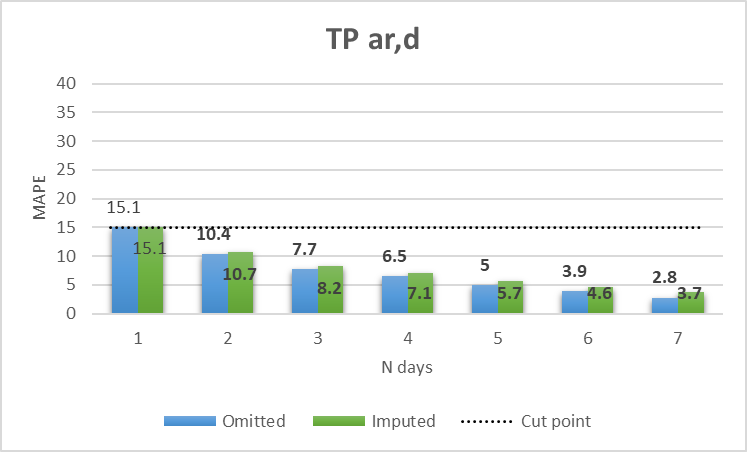 | 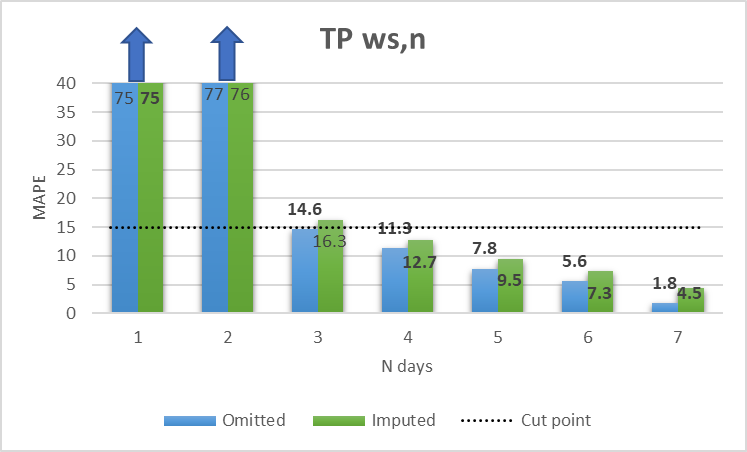 |
| 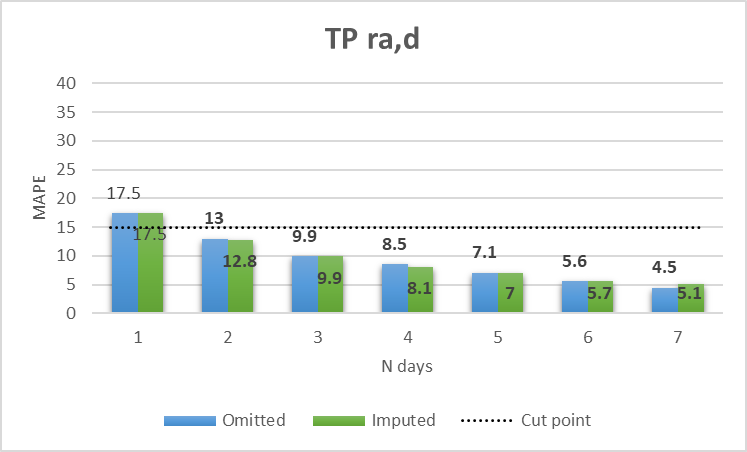 | 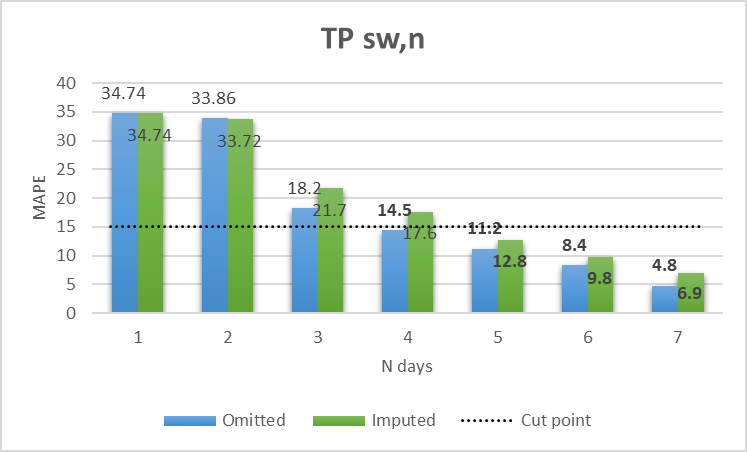 |
| 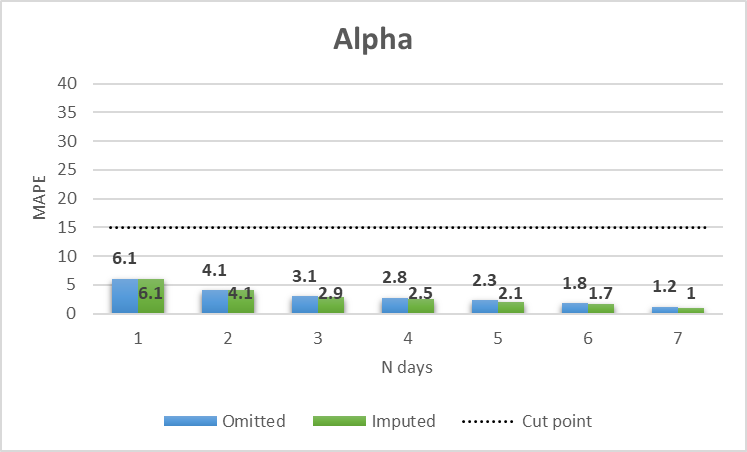 | 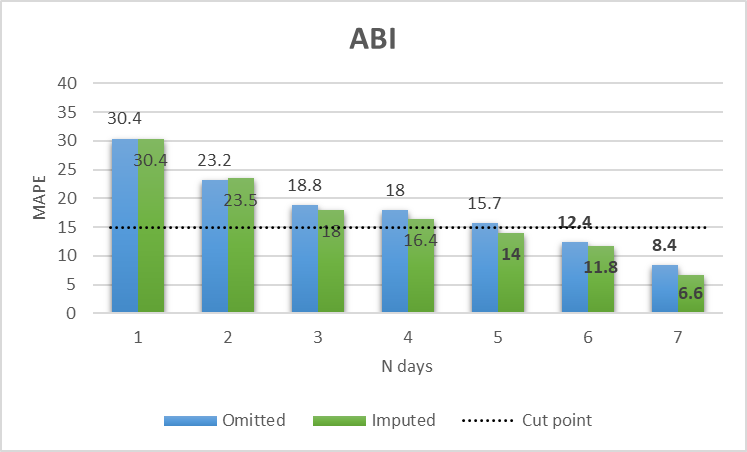 |
| **Abbreviations:** mean absolute percent error (MAPE), inter-daily stability (IS), intradaily variability (IV), transition probability (TP), TP from activity to rest during the day (TP_ar,d_), TP from wake to sleep during the night (TP_ws,n_), TP from rest to activity during the day (TP_ra,d_), TP from sleep to wake during the night (TP_sw,n_), and activity balance index (ABI).  Bold values correspond to MAPE≤15%  * An entire day is defined as the period between wake up to next wake up (it combines the day (wake up to sleep onset) and night (sleep onset to wake up) periods). | |

| Figure 14 Illustration of the impact of imputation of a non-wear period… | |
| --- | --- |
| **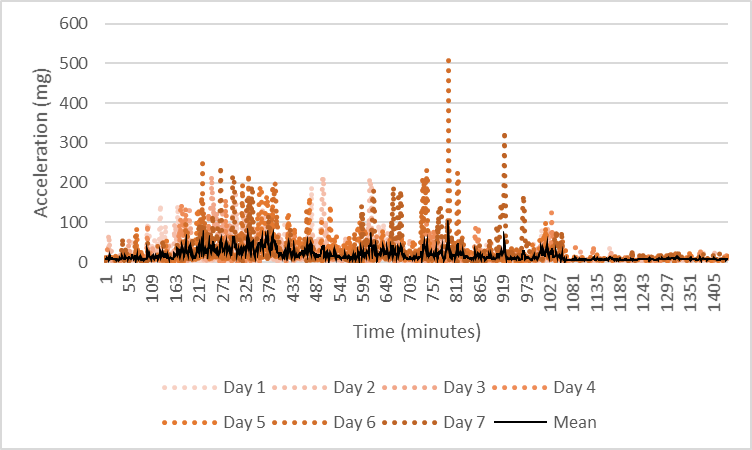**  **(a)** | **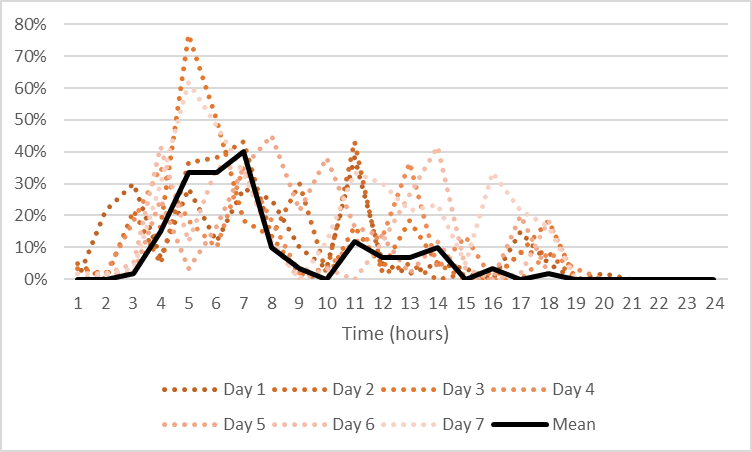**  **(b)** |
| **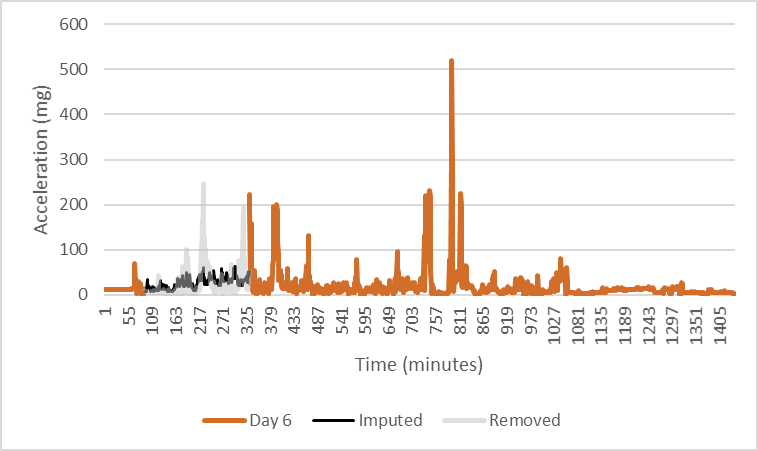**  **(c)** | **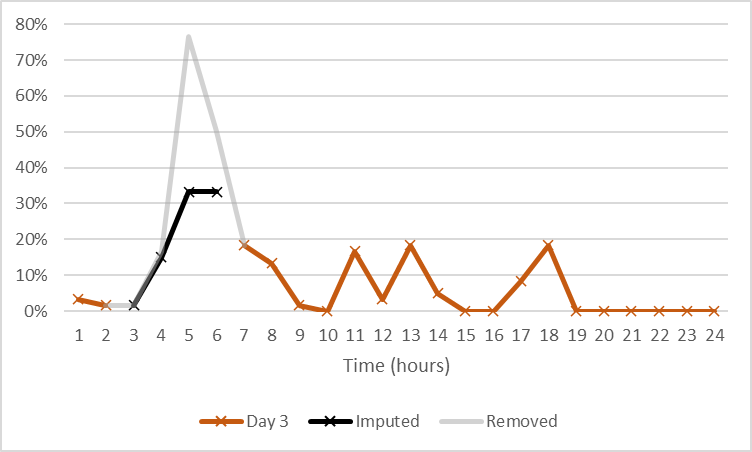**  **(d)** |
| **Note:** This graph shows real data for: **(a)** acceleration per minute in milligravity (mg) of one individual per time of the day for seven days, **(b)** percentage of activity (one-minute epoch mean acceleration≥40 m*g*) per hour of the day for seven days, **(c)** acceleration per minute of the Day 6 with four hours of imputed data, **(d)** percentage of activity per hour of Day 3 with four hours of imputed data.  We observe that the imputed signal tends to underestimate the variability of the time series (and overestimate the similarity). Thus, the IS metric (which is a measure of similarity) corresponding to this over-smoothed time series is higher than the IS corresponding to the original time series. | |
